# Common features of rare disease patients in the emergency department: a systematised literature review

**DOI:** 10.1186/s13023-025-04111-6

**Published:** 2025-11-13

**Authors:** Sandra Pflock, Hannah Carolina Mücke, Rajan Somasundaram, Eva Diehl-Wiesenecker

**Affiliations:** Department of Emergency Medicine, Campus Benjamin Franklin, Charité – Universitätsmedizin Berlin, Corporate Member of Freie Universität Berlin and Humboldt-Universität zu Berlin, Hindenburgdamm 30, 12203 Berlin, Germany

**Keywords:** Emergency department, Rare diseases, Inborn errors of metabolism, Acute hepatic porphyria, Emergency physician, Common features

## Abstract

**Background:**

Patients with rare diseases (RD) may present to the emergency department (ED) with an acute manifestation of an undiagnosed RD. If not identified, some of these patients may suffer from increased morbidity, mortality and worse outcomes compared to patients with more common diseases. Unfortunately, patients with RD often present with common symptoms, e.g. abdominal pain, making it even more difficult for ED physicians to identify those patients. Thus, strategies are needed to determine when certain RD should be suspected in the ED. We aimed to identify common features of RD through a systematised literature review of a predefined set of RD.

**Methods:**

Embase and MEDLINE were searched for eligible studies published in all years up to November 2023. Studies reporting clinical characteristics of patients who presented to the ED with one out of a predefined set of eight RD were included: acute hepatic porphyria, Fabry disease, familial Mediterranean fever, hereditary angioedema, hereditary hemorrhagic telangiectasia, myasthenia gravis, paroxysmal nocturnal hemoglobinuria and thrombotic thrombocytopenic purpura. Clinical characteristics, symptoms and family history were extracted to explore potential common features. Database searches, title, abstract and full-text screening and data extraction were conducted by a single reviewer.

**Results:**

Of the 4 732 articles identified, 18 were included in the review. For two RD no articles and for three RD only one article could be included. All but two prospective studies were retrospective chart reviews or case series reporting on small patient cohorts (*n* = 3 - 75 patients). The common features of patients were: age under 65 years, recurrent symptoms affecting multiple organ systems, normal or nonspecific findings in routine laboratory tests and imaging, presence of trigger factors and a positive family history of similar symptoms or RD.

**Conclusion:**

This review highlights the limited knowledge about RD in the ED setting. Some common features of RD patients were identified that could aid future research in developing risk stratification algorithms to facilitate early detection and treatment of RD in the ED. To our knowledge, this is the first literature review to explore the common features of patients with RD in the ED.

**Supplementary Information:**

The online version contains supplementary material available at 10.1186/s13023-025-04111-6.

## Background

Emergency departments (ED), and thus, emergency medicine, provide care for patients requiring diagnosis and management of urgent and emergency aspects of illness and injury with a full spectrum of undifferentiated physical and behavioral disorders [1]. Therefore, ED are often the first place for patients with acute symptoms, including patients with certain (unknown) rare diseases (RD).

There is no global definition for RD [2]. A disease is considered rare, if it affects no more than five out of 10 000 people in the EU [3], less than 200 000 in the US [4] or less than 50 000 in Japan [5]. Given that more than 7 000 RD have been described [6], RD may affect up to 36 million people in the EU [7] and up to 446 million people worldwide [8].

Since a huge number of patients (140 million in the US [9], 19 million in Germany [10] or 21 million in France [11], respectively) visit ED annually, it might be reasonable for ED physicians to consider RD during clinical decision making, particularly in ED patients with otherwise unexplained symptoms.

Patients with RD face problems, inside and outside the ED, regarding diagnosis and treatment resulting in diagnostic latency, misdiagnosis and multiple healthcare contacts before diagnosis [12, 13]. On average, RD patients wait 4.7 years [14] to receive a correct diagnosis. Compared with patients with more common diseases, they experience increased morbidity and mortality, both overall and in the ED [15–17] and worse inpatient outcomes [18].

Strategies are needed to determine when to suspect an RD, as a lack of suspicion seems to be a major factor in diagnostic delay [19]. Unfortunately, patients with RD present with common symptoms, e.g. abdominal pain, making it even more difficult for ED physicians to identify those patients. Recent studies have indicated the need for emergency specific education on RD [17, 20] and have started to provide strategies for RD in the ED [21–23]. The feasibility of screening for RD in the ED setting has been demonstrated [24–26] and the number of therapies (“orphan drugs”) available is increasing [27]. Therefore, the prognosis of patients with RD depends crucially on early diagnosis and initiation of treatment.

“Red flags” could help to identify RD patients [28]. To explore potential common features among patients with RD in the ED that could serve as such, we conducted a systematised literature review.

## Methods

In the first step, a set of diseases was defined to represent RD most relevant for identification in the ED because a) patients experience acute symptoms in adolescence or adulthood and are relevant for ED physicians beyond paediatrics, b) initial diagnostic tools in the ED can rule out or include a rare differential diagnosis and c) treatment options for the initial management in the ED or prognostic relevant long-term therapies exist, making the diagnosis in the ED urgent. PubMed and Orphanet were searched on the basis of these criteria to identify candidate RD. The final set, agreed upon by all the authors, encompassed eight RD: acute hepatic porphyria (AHP), Fabry disease (FD), familial Mediterranean fever (FMF), hereditary angioedema (HAE), hereditary hemorrhagic telangiectasia (HHT), myasthenia gravis (MG), paroxysmal nocturnal hemoglobinuria (PNH) and thrombotic thrombocytopenic purpura (TTP). A brief description of each RD and its emergency specifics is provided in Table 1. The set is not exhaustive and many more RD fall within these criteria.

**Table 1:** Selected set of rare diseases in adults that may be seen in the ed and for which a treatment option exists

	Epidemiology: typical age of onset, gender distribution and estimated prevalence	Pathophysiology and clinical manifestation	Emergency Department (ED) management, treatment options
AHP	**Manifestation**: after puberty W > M **Prevalence**: ~1/100.000 [29–31]	Inborn error of metabolism (IEM): Inherited enzyme dysfunction in hepatic heme biosynthesis pathway leads to accumulation of porphyrin precursors (aminolevulinic acid and porphobilinogen). Described trigger: medication, menstrual cycle, fasting, stress, infection. Acute attack(s): neuropsychiatric (neuropathic limb, back or chest pain, paresis, confusion), gastrointestinal (severe abdominal pain, constipation, nausea) and cardiovascular (hypertension, tachycardia) symptoms to severe complications (hyponatremia, seizures, tetraplegia, respiratory paralysis). Chronic symptoms may occur between attacks. [32, 33]	**Diagnostic approach in ED:** Aminolevulinic acid and/or Porphobilinogen increased fourfold or higher in spot urine (sample must be stored light protected). **Therapy:** **ED management:** elimination of aggravating factors, pain medication (often opioids necessary), antiemetics, glucose, hemin. Approved since 2019 (FDA)/2020 (EMA): small interfering RNA (siRNA) Givosiran for reduction of acute attacks and chronic symptoms. [34, 35] [32]
FD	**Manifestation:** Mainly affects males (between 3 and 10 years), but females too (6–15 years) First event (TIA, stroke, cardiac event, CKD stage 5) in classical FD at age of 41–50 years [36, 37] **Prevalence**: 1/40.000 to 1/117.000 live births [38–40]	IEM: X-Chromosomal inherited lysosomal storage disease. Defect in GLA-gene causes inactivity (classic form) or reduced activity (late-onset form) of lysosomal enzyme alpha-Galactose A (AGLA). Affected organs and related symptoms vary widely between patients, e.g.: heart (left ventricular hypertrophy, arrythmias, infarct), kidney (proteinuria, CKD), peripheral and central nervous system (neuropathic pain, fatigue, stroke, TIA), gastrointestinal tract (pain, constipation, diarrhea, nausea), ears (tinnitus, sensorineural hearing loss), skin (angiokeratoma, reduced or excessive sweating). [41, 42]	**Diagnostic approach in ED**: Dried blood spot card to determine AGLA-activity (men). In women a pathologic variant in GLA-gene needs to be examined. **Therapy:** **ED management**: Treatment of stroke, heart failure, pain, myocardial infarct, arrhythmias. [43] Enzyme replacement therapy (Agalsidase alfa, Agalsidase beta), oral chaperone therapy (Migalastat). [42]
FMF	**Manifestation:** in first decade, ~5% onset after age of 30. [44, 45] W = M up to M > W (3:2) [46] **Prevalence**: Common in Mediterranean and middle eastern region (e.g. Turkey 1:400 to 1:1000, Israel 1:1000, Armenia 1:500, Jordan, Lebanon, Syria), rare in non-Mediterranean regions. [47]	Autoinflammatory disease, most common periodic fever syndrome. Recurrent acute attacks (lasting 1–3 days) of fever with possible manifestations of serous membranes (abdominal pain, chest pain), skin (erysipelas-like erythema) and joints (arthralgia, arthritis) due to dysregulation of innate immune system caused by defect in MEFV gene (mostly autosomal recessive inherited). [46, 48, 49]	**Diagnostic approach in ED:** clinical criteria (Tel Hashomer, Livneh, Eurofever/PRINTO), raised CRP [50] **Therapy:** **ED management**: determine CRP, supportive care consisting of sufficient pain management (NSAID, paracetamol, metamizole; opioids can be necessary) and intravenous hydration, consider/refer to specialist care for consideration of Glucocorticoids, IL-1 blockers [51–53] Colchicin, Biologicals (IL-1 blocker, TNF inhibitors) to prevent/reduce attacks and prevent long term complications (e.g. AA-amyloidosis) [54]
HAE	**Manifestation**: in childhood or adolescence (first or second decade of life) W = M **Prevalence**: 1.1–1.6/100.000 [55, 56]	Bradykinin-mediated angioedema: due to inherited enzyme defect. In most cases, a quantitative or functional deficiency of the C1-esterase inhibitor (C1-INH) leads to oversupply of bradykinin causing angioedema attacks. HAE – attack: painful recurrent swelling of the face, tongue, throat, larynx, extremities and/or genitals; visceral angioedema can cause abdominal pain with nausea and vomiting. No urticaria. [22, 56]	**Diagnostic approach in ED**: Activity and concentration of C1-INH and concentration of C4 complement in serum or plasma. **Therapy:** **ED management**: evaluation for airway involvement and airway management if necessary, C1-INH concentrate (intravenous or subcutaneous), Bradykinin receptor antagonist; solvent detergent plasma or fresh frozen plasma if specific treatment is not available. Long term prophylaxis (C1-INH, lanadelumab, Berotralstat), short term prophylaxis before potential HAE inducing events (e.g. surgical or dental procedures) and on-demand treatment with C1-INH or bradykinin receptor antagonist (or kallikrein inhibitor). [57]
HHT	**Manifestation**: of epistaxis by about half of patients < 20 years of age. [58] W = M **Prevalence**: 1–2/10 000 [59]	Vascular disease: autosomal dominant inherited disorder of the connective tissue of the blood vessels causing mucocutaneous telangiectasia (located on: lips, oral cavity, fingers, nose) and visceral arteriovenous malformations (gastrointestinal telangiectasia (with or without bleeding), pulmonary AVM, hepatic AVM, cerebral AVM, spinal AVM). [60] Recurrent spontaneous Epistaxis is the most common symptom. [58]	**Diagnostic approach in ED**: clinical criteria (Curaçao Criteria) [60] **Therapy:** **ED management**: Epistaxis management (consider complications due to recurrent/severe epistaxis such as anemia, hemodynamic instability/shock, chronic cardiac failure), management of complications of pulmonary or cerebral AVMs (stroke, brain abscess, haemorrhage). [61–63] Epistaxis management (topical saline, oral tranexamic acid, ablative therapies, bevacicumab), management of iron deficiency and anemia, screening for and treatment of relevant AVMs to avoid unnecessary stroke and life-threatening hemorrhage. [64]
MG	**Manifestation**: incidence increases with age in both sexes with a peak between the 6th and 8th decade of life. [65] Bimodal incidence peak in women (20–40 years and > 70 years), Men > Women in late onset MG ( > 50 years) [65] **Prevalence**: 5.35–46.5/100,000 [66–68]	Autoimmune disease: autoantibody-caused impaired transmission at neuromuscular junction resulting in use related weakness of affected muscles. Typical symptoms include (asymmetric) diplopia, ptosis, bulbar muscle involvement (e.g. difficulties swallowing), weakness of proximal muscles and respiratory weakness. [69] In approximately 50% of patients MG manifests with ocular symptoms (diplopia, ptosis) and progresses to generalised MG in up to 80% mostly within the next two years. [70, 71] Life-threatening complication of MG is myasthenic crisis. Patients with myasthenic crisis are at risk of respiratory failure and therefore need to be closely monitored (rapid progression with need for intubation possible). [72] Red flags suggesting possible myasthenic crisis are: history of febrile infection treated with antibiotics in the last two weeks, difficulties in swallowing and speech, dropping of head or chin. [73]	**Diagnostic approach in ED**: Anamnesis and physical examination: use related muscle weakness. Ice-pack test as bedside test option in the ED (76% sensitivity, 98% specificity). [74] **Therapy:** **ED management of myasthenic crisis**: close monitoring of respiratory function, admission to IMC or ICU, intravenous immunoglobulin/plasma exchange therapy. Acetylcholinesterase inhibitors (e.g. pyridostigmine), glucocorticoids and immunosuppressants (e.g. azathioprine), thymectomy, complement inhibitors (eculizumab, ravulizumab), rituximab based on patients age, thymic pathology, antibody status and disease activity. [75]
PNH	**Manifestation**: median age 30–40 years W = M up to W > M [76–78] **Prevalence**: ~ 5–16/Mio [77, 79]	Hematopoetic disease: acquired defect of hematopoetic stem cells (somatic mutation in PIGA gene causing GPI anchor defect with changes in surface proteins) causing chronic complement-mediated hemolysis, (pan-)cytopenia and increased risk for thrombosis. [80] Variable unspecific signs and symptoms: signs of anemia (e.g. fatigue, headache, shortness of breath), thrombosis (main cause of death), hemoglobinuria, abdominal pain, chest pain, dyspnoea, dysphagia, erectile dysfunction, impaired renal function. PNH patients may have a history of bone marrow disease (e.g. aplastic anemia). [76, 78, 81]	**Diagnostic approach in ED**: Coombs-negative hemolytic anemia, (pan-)cytopenia, thrombosis (typical or atypical location, in combination with cytopenia and/or hemolytic anemia, unprovoked/no risk factors present). (PNH clone cells in flow cytometry.) [82] **Therapy:** **ED management**: Treatment of PNH-related emergencies: - thrombosis (life-time risk of 50%): can occur in typical (extremities) or atypical (e.g. Budd-Chiari syndrome, mesenteric or portal veins, cerebral veins, dermal veins) locations; tendency for thrombosis in abdominal or/and intracranial veins; can even occur under adequate anticoagulative therapy - severe pancytopenia - severe haemolytic crisis - abdominal pain crisis [83, 84] Long-term treatment can increase to normalise life expectancy: Eculizumab (C5 inhibitor), Ravulizumab (long-acting C5 inhibitor), Pegcetacoplan (C3 inhibitor). Patients treated with complement inhibitors have an increased risk for infections with capsule forming bacteria (e.g. meningococci, pneumococci). [77]
TTP	congenital TTP: hereditary, W = M, manifestation in early childhood or adulthood immune mediated TTP: acquired, adults in 3rd-5th decade, W > M (2.5:1 to 3.5:1) annual incidence of iTTP in Europe: 1.5 - 6.0/1 Mio. [85–87]	Thrombotic microangiopathy: inherited or acquired deficiency of ADAMTS-13 leading to platelet rich thrombi causing thrombocytopenia, microangiopathic haemolysis and ischaemic organ dysfunction up to organ failure presenting with neurological symptoms (headaches, confusion, delirium, coma, paresis, visual disturbances, seizures, speech or language disorders), signs of thrombocytopenia/anemia, sometimes acute kidney damage or fever. Further end organ damage, e.g. to heart (troponinaemia), lungs, pancreas or GI tract can occur. Primary iTTP where no underlying factor (disease, trigger) is evident accounts for the majority of TTP cases. [88–91]	**Diagnostic approach in ED**: Thrombocytopenia + hemolytic anemia. Fragmentocytes in blood smear. ADAMTS13 antibodies and determination of ADAMTS13 activity (TTP < 10%). PLASMIC score and French score. **Therapy:** **ED management**: Therapeutic plasma exchange, glucocorticoids, caplacizumab, rituximab. Regular ambulatory monitoring of ADAMTS13 activity/antibody and blood count/hemolysis parameters. Prophylaxis with recombinant ADAMTS13 in patients with cTTP. [92–94]

AHP – acute hepatic porphyria, FD – Fabry disease, FMF – familial Mediterranean fever, HAE - hereditary angioedema, HHT - hereditary hemorrhagic telangiectasia, MG - myasthenia gravis, PNH - paroxysmal nocturnal hemoglobinuria, TTP - thrombotic thrombocytopenic purpura, w – women, *m* – men, IEM – inborn error of metabolism, EMA - European Medicines Agency, FDA - U.S. Food and Drug Administration, siRNA – small interfering RNA, TIA - Transient ischemic attack, CKD - Chronic kidney disease, NSAID - non steroidal anti-inflammatory drug, AVM - arteriovenous malformations

In the second step a systematised literature review [95] was conducted to investigate the common and distinct features of the predefined set of RD in the ED. The search strategy was developed and discussed collectively by all the authors. Title and abstract screening, full-text evaluation and data synthesis were performed by a single reviewer. In cases of uncertainty of eligibility of a study, two additional reviewers assessed the full text and resolved the inclusion decision by discussion and consensus. The review adhered to the principles outlined in the Joanna Briggs Institute (JBI) Manual for Evidence Synthesis Chap. 5 and reporting followed the Preferred Reporting Items for Systematic Reviews and Meta-Analyses (PRISMA) 2020.

### Selection of diseases

The selection of the eight RD was made by a panel of three experienced emergency physicians based on their professional judgment. Given that a comprehensive literature review across the entire spectrum of RD was not feasible, these eight conditions were selected as illustrative examples. This approach enabled a focused literature review, while acknowledging that the list is neither systematic nor exhaustive. Rather, it was intended to highlight a diverse range of presentations and to facilitate the identification of recurring features across different disease entities. The defined set should therefore be understood as exemplary of a wider group of RD meeting the above criteria.

### Search strategy

Embase and MEDLINE were searched in November 2023 without restrictions on the publication period. The search string comprised a combination of various synonyms for the ED and each RD within the described set, connected by Boolean operators (OR and AND). The complete search strategy is presented in Supplement 1.

### Inclusion criteria

The research question and inclusion criteria were defined according to the CoCoPop strategy [96]:

- Condition: AHP, FD, FMF, HAE, HHT, MG, TTP, PNH.
- Context: All types of emergency departments (e.g. urban, rural, academic, community).
- Population: Patients 16 years or older.

We included all studies reporting primary data, including case series, published up to November 2023. No restriction on publication date range was applied. Outcomes of interest were patient characteristics (demographics, clinical status, medical history), disease presentation (symptoms, affected organ systems, presenting symptom in ED), risk factors (trigger factors, recent new medication, recent pregnancy), clinical assessment (vital signs, laboratory results, physical examination findings, imaging results) and clinical course in the ED (diagnostic process, ICU admission, total ED visits, death).

### Exclusion criteria

The following exclusion criteria were applied: setting other than the ED, age < 16 years, RD other than the defined set, inappropriate publication type (case report, review, letter to the editor, conference abstracts, conference reviews). Only studies in English and German were included, as the team is proficient in these languages. If no setting was specified, the study was excluded. All studies were included regardless of sample size, ensuring a comprehensive review of available evidence. Studies that did not mention any of the patient characteristics according to the outcomes of interest described above were excluded from the review.

### Selection of articles

All eligible articles from MEDLINE were exported, whereas for EMBASE, a filter was applied to exclude unsuitable publication types (conference abstracts, conference reviews, conference papers, notes, errata, letters to the editor). Case reports and reviews were not automatically excluded. For deduplication and title and abstract screening the free version of the Rayyan.ai web application was used. Rayyan.ai is an AI-powered tool registered in Qatar designed to support the process of systematic literature reviews by facilitating the screening and management of research articles [97, 98]. Full texts whose title and abstract appeared suitable or whose suitability could not be adequately assessed on the basis of the abstract (e.g. no abstract available) were retrieved and evaluated on the basis of the defined inclusion and exclusion criteria. The reasons for exclusion were documented for each publication. The initial screening and selection of studies was conducted by a single reviewer. In cases of uncertainty regarding the eligibility of a study, two additional reviewers were consulted. These reviewers assessed the full text of the respective publication and reached a consensus on inclusion or exclusion through discussion and consensus.

### Data extraction

An adapted version of the JBI data extraction form for prevalence studies was used to gather patient and RD characteristics in the ED, including age, sex, age of manifestation of RD, disease history, all reported symptoms, presenting symptoms in the ED, family history, vital signs, laboratory, physical examination, (incorrect) differential diagnoses made, number of visits to the ED, trigger factors, pregnancy, admission to the ICU, death and other findings of interest. Missing data were documented as N/A. A single reviewer extracted data from all included studies. The extracted data was then presented to two additional reviewers for verification and discussion. Throughout the extraction process, regular team meetings were held to discuss any challenges or ambiguities encountered and to maintain consistency in data interpretation.

### Quality assessment

This review focused on reported symptoms, clinical features, family history and patient characteristics of adult and adolescent patients presenting to the ED. Therefore, all studies that reported patients diagnosed with one disease of the defined set of RD in the ED context were included if the study met the inclusion and exclusion criteria, even if the study sample size was small or if the description of the study subjects was inconclusive. No further systematic quality assessment was performed.

### Data synthesis

Owing to the expected heterogeneity and limited number of publications, the findings were synthesised in a narrative form. For each RD, separate tables were created, collecting and summarising all information that could be extracted from the studies using the data extraction form. The findings were thematically grouped and structured in comparative tables to make key features for each RD clearly visible. Data were first condensed and analysed separately for each RD and then across all RD, to identify recurrent patterns. The tables were compared to detect common features. In addition to the outcomes, study characteristics, including number, age and gender of participants, number of studies for each RD, study design and extractable outcomes, were compiled in a comparative table to facilitate assessment of the robustness of results.

## Results

### Study selection

**Fig. 1:**
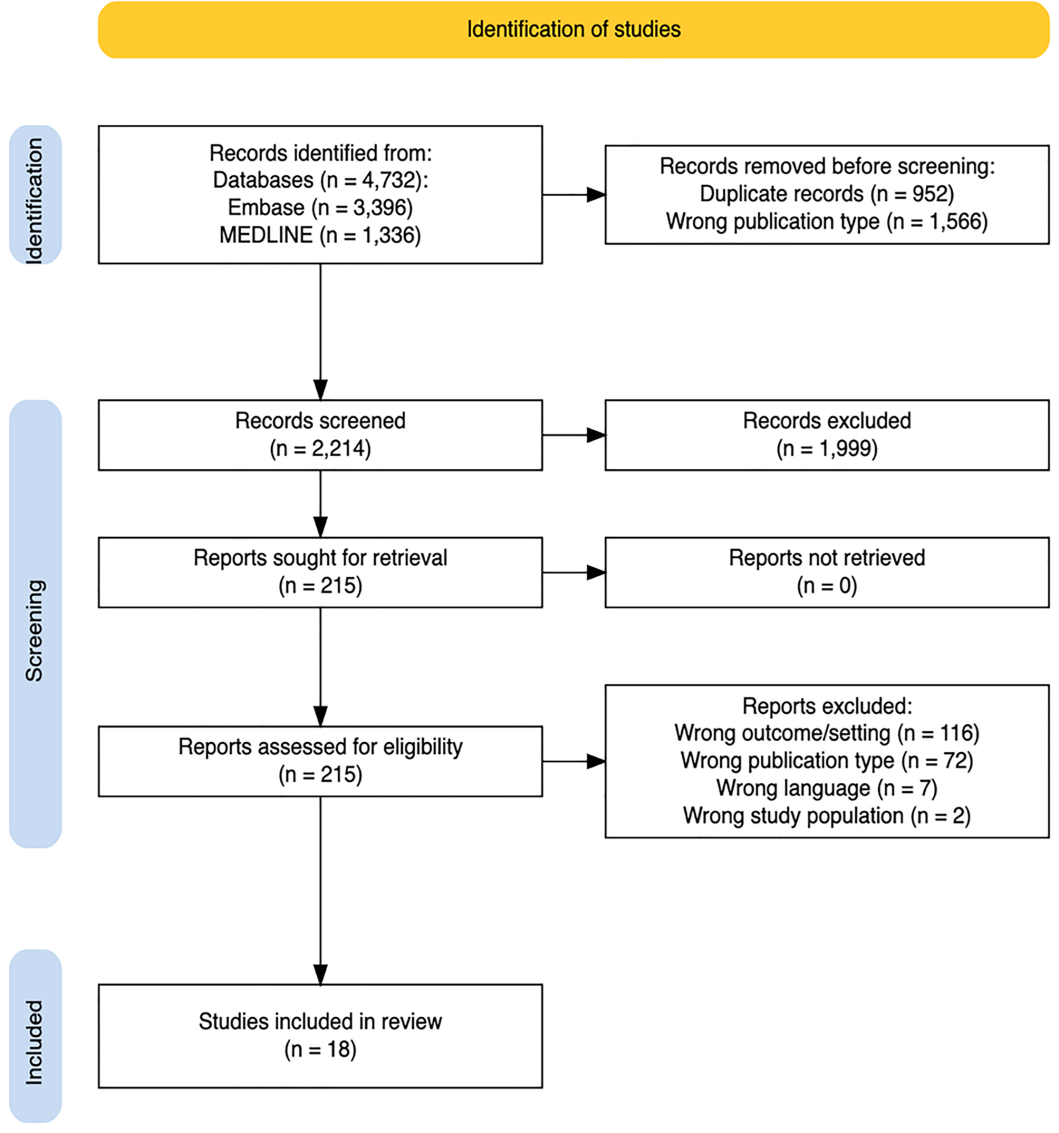
Prisma (preferred reporting items for systematic reviews and meta-analyses) flow diagram [99]

In total, 4.732 records were identified in Embase and MEDLINE (Fig. 1). Ineligible report types were automatically excluded by filtering (letter to the editor, conference abstract, conference paper, conference review, erratum, note). After deduplication, 2 214 records were assessed for title and abstract screening. The title and abstract were considered ineligible (*n* = 1 999) if they obviously did not intend to describe the characteristics of patients aged 16 years or older with one of the eight defined RD, if the study population did not present to an ED, if it was a case report or review or if the language was other than English or German. The remaining 215 reports were considered for the full-text screening. All full texts were evaluated on the basis of the predefined inclusion and exclusion criteria. Most of the assessed reports (*n* = 116) were excluded because they either did not describe patient characteristics or the assessed patients had not presented to an ED or both [100–102]. For studies reporting on both ED patients and patients who presented elsewhere [103], only ED patients’ outcomes were retrieved. The publication type was considered inapplicable, if it was a case report or review (*n* = 72). Two full texts were excluded for assessing patients aged < 16 years and another seven were excluded for being in a language other than English or German. Excluded full texts and the reasons for exclusion are listed in Supplement 2. Thus, 18 articles were left for inclusion [25, 72, 103–118]. No studies could be included for PNH and FD, despite the low-threshold inclusion and exclusion criteria.

### Study characteristics

The study’s characteristics are presented in Table 2. Among the 18 included studies, 11 were retrospective chart reviews with small sample sizes, ranging from 4 to 36 patients. One study [109] included patients with HAE only as a subgroup and did not report their size. Four studies [72, 111, 112, 114] were case series, each describing only three patients, for MG and HHT being the only identified articles reporting on clinical patient characteristics in the ED. Three prospective observational studies were included for HAE and FMF (3 to 75 described patients) [25, 107, 110].

**Table 2:** Study characteristics

RD	Authors (year), country	Study design, No. of described patients	Patient Age (years)	Patient gender (female)	RD already known	Study objective	Outcome (described patient/disease characteristics)
AHP	Kumar et al. (2010), India	RCR, *n* = 13	mean 25.7 ± 14.9	5/13	Yes 7/13	Characterisation of acute intermittent porphyria presentation in the ED.	patient age (years), patient gender, age of disease onset, history of RD, all reported symptoms, family history, vital signs (initial), laboratory, imaging, trigger factors, death
	Liu et al. (2005), Taiwan	RCR, *n* = 10	N/A	7/10	No	Characterisation of AHP presentation in the ED.	patient age (years), patient gender, age at disease onset, history of RD, presenting symptom in ED, family history, laboratory, physical examination, total ED visits
	Yang et al. (2016), China	RCR, *n* = 36	Median 25.3 (range 18–45)	33/36	N/A	Characterisation of AHP presentation in the ED.	patient age (years), patient gender, all reported symptoms, vital signs (initial), laboratory, differential diagnosis/other applied diagnosis in ED, trigger factors, recent new medication, death
FMF	Huseyin et al. (2014), Turkey	prospective two-site observational study, n = 75	mean: 38.7 ± 12.6 and 33.1 ± 12.1 (2 groups)	41/75	Yes	Investigation of regional and ethnic differences in trigger factors of FMF.	patient age (years), patient gender, history of RD, presenting symptom in ED, family history, trigger factors
HAE	Day et al. (2023), Southafrica	RCR, *n* = 11	Study population: median 57 (IQR 43 - 67) HAE-Subgroup: NA	10/11	Yes	Characterisation and outcome of acute angioedema in ED.	patient age (years), patient gender, history of RD, all reported symptoms (regarding ED visit), presenting symptom in ED, trigger factors
	Felder et al. (2014), Canada	RCR, *n* = 455, HAE-subgroup NA	Study population: mean 37.8 (SD 22.8) HAE-Subgroup: NA	Study population: 60.6%, HAE-subgroup: NA	Yes	Characterisation of undifferentiated angioedema in ED.	history of RD, all reported symptoms (regarding ED visit), presenting symptom in ED
	Moldovan et al. (2018), Romania	Case series, n = 3	42, 52, 59	1/3	Yes 2/3	Case series to demonstrate fatal outcome in missed or mismanaged laryngeal angioedema in HAE patients.	patient age (years), patient gender, age at disease onset, history of RD, all reported symptoms (regarding ED visit), family history, death
	Hirose et al. (2017), Japan	prospective multicentre observational screening study, *n* = 3	median 52 (IQR 38–85)	2/3	No	Determine incidence of HAE patients in ED.	patient age (years), patient gender, age at disease onset, history of RD, all reported symptoms (regarding ED visit), presenting symptom in ED, family history, laboratory, Imaging, differential diagnosis/other applied diagnosis in ED, total ED visits/history of ED visits, ICU admission, death
	Javaud et al. (2015), France	prospective multicentre observational study, *n* = 29	median 29 (IQR 26–36)	19/29	Yes	Identify factors associated with hospital admission in HAE patients presenting to ED with acute attack.	patient age (years), patient gender, history of RD, presenting symptom in ED, trigger factors, ICU admission
	Pekdemir et al. (2007), Turkey	Case series, n = 3	21, 33, 35	all	Yes 2/3, NA 1/3	Presentation of HAE patients treated with fresh frozen plasma.	patient age (years), patient gender, age at disease onset, history of RD, presenting symptom in ED, family history, Vital signs (initial), total ED visits/history of ED visits, trigger factors, death
	Songur Kodik et al. (2023), Turkey	RCR, *n* = 35	mean 36.4 (SD 13)	54.3%	Yes and no, exact number N/A	Calculate LACE index and identify factors to predict recurrent admission in HAE patients.	patient age (years), patient gender, age at disease onset, history of RD, presenting symptom in ED, family history, Vital signs (initial), total ED visits/history of ED visits, trigger factors, death
HHT	Kuwayama et al. (2003), Japan	Case series, n = 3	23, 32, 62	1/3	No	Presentation of three HHT cases with neurological emergency.	patient age (years), patient gender, history of RD, presenting symptom in ED, family history, laboratory, physical examination, Imaging, differential diagnosis/other applied diagnosis in ED
MG	Smulowitz et al. (2005), USA	Case series, n = 3	48, 78, 65	all	Yes 1/3	Characterisation of MG presentation in the ED.	patient age (years), patient gender, age of disease onset, history of RD, all reported symptoms, presenting symptom in ED, Vital signs (initial), physical examination, Imaging, death
TTP	Boisriou et al. (2023), France	Multicentre RCR, *n* = 40, TTP - subgroup = 29	Median 44 (IQR 28–57)	27 (67.5%)	No	Misdiagnosis of Thrombotic microangiopathy in the ED.	patient age (years), patient gender, age of disease onset, history of RD, all reported symptoms, presenting symptom in ED, laboratory, differential diagnosis/other applied diagnosis in ED, Recent pregnancy, death
	Li et al. (2021), China	RCR, *n* = 19	Median 56 (Range 19 - 75)	10/19	No	Characterisation of TTP presentation in ED.	patient age (years), patient gender, age of disease onset, history of RD, all reported symptoms, presenting symptom in ED, laboratory, trigger factors/aetiology, death
	Noel et al. (2013), France	RCR, *n* = 12, TTP-subgroup = 6	Median 35.2 (range 23.4–74)	4/6	N/A	Discriminate TTP and pseudothrombotic microangiopathies related to vitamin B12 deficiency (pseudo- TMA).	patient age (years), patient gender, all reported symptoms, laboratory, death
	Pieralli et al. (2011), Italy	RCR, *n* = 12	mean 59 (range 37—85)	58%	No	Factors to predict in-hospital death of TTP.	patient age (years), patient gender, age of disease onset, history of RD, all reported symptoms, presenting symptom in ED, vital signs (initial), laboratory, trigger factors/aetiology, recent pregnancy, death
	Stella et al. (2009), USA	Multicenter RCR, *n* = 12, ED - subgroup = 4	range 20–40	all	Yes 1/12	Characterisation of acute presentation of TTP in ED and obstetric triage in pregnancy.	patient age (years), patient gender, age of disease onset, history of RD, all reported symptoms, presenting symptom in ED, vital signs (initial), laboratory, differential diagnosis/other applied diagnosis in ED, total ED visits, recent pregnancy, death

RCR = retrospective chart review, N/A – not known/no information

Half of the studies were conducted in East Asia (*n* = 5) (China, Taiwan, Japan), India (*n* = 1) and Turkey (*n* = 3), the other half in Europe (*n* = 5) (France, Italy, Romania), North America (*n* = 3) (USA, Canada) and one study in South Africa. Although there were no restrictions on the study period, the included studies were published between 2003 and 2023.

### Risk of bias in studies

Only half of the study objectives were designed to characterise patients and their RD in the ED. The deviating focus limited the characterisation. As expected, studies reported very different on patient characteristics and to varying extents. Four of the 18 studies were case series selectively reporting on patients individually selected by the authors. Therefore, very selective and mostly small patient cohorts formed the basis of this review.

### Patients with acute hepatic porphyria (AHP) in the ED

Three studies [104–106] described the clinical presentation of 59 patients with AHP aged 16 years or older in the ED (Table 3).

**Table 3:** Characteristics of ahp patients in the ed

	Kumar et al. 2010	Liu et al. 2005	Yang et al. 2016
Patients (female, male)	n = 13 (f: 5, m: 8) *only patients with AIP*	n = 10 (f: 7, m: 3)	n = 36 (f:33, m:3)
Age (years)	mean 25.7 ± 14.9	N/A	Median 25.3 (range: 18–45)
AHP known	Yes 7/13	No	N/A
Age of AHP onset	17–37 (12/13 patients with manifestation before age of 30 years)	17–55 (mean age: 32)	N/A
Symptoms (No. of patients)	**Gastrointestinal:** - abdominal pain (11/13) - vomiting (8/13)	**Gastrointestinal:** - abdominal pain (10/10)	**Gastrointestinal:** - abdominal pain(32/36) - constipation (26/36)
	**Neurological:** - seizure (7/13) - acute quadriparesis or quadriplegia (7/13) - peripheral neuropathy (4/13) - respiratory paralysis (1/13)	**Neurological** - muscle weakness (1/10) - seizure (2/10, only after inpatient admission)	**Neurological** - confusion 16/36 - seizure 12/36 - paresis 5/36 - numbness of extremities 3/36 - respiratory paralysis 2/36
	**Other:** temporary red urine in history (3/13)	**Other:** dark urine (1/10)	cyclic attacks 28/36
Reason for ED visit	N/A	abdominal pain (10/10)	N/A
Vital signs	hypertension 4/13	N/A	tachycardia 10/36 hypertension 3/36
family history of AHP	0/13	2/10	N/A
trigger factors	medication (7/13), infections	N/A	28/36 cyclic attacks linked to menstruation cycle 2/36 history of alcohol consumption 3/36 history of severe dieting 1/36 valproat
Additional findings	Ultrasound (abdomen), MRI or CT (head) were either normal or not performed (except in one patient: bilateral parietoocipital infarct area).	hyponatremia 3/10 normal: WBC, CRP, liver enzymes, lipase, amylase patients required at least 2 different painkillers (mean: 2.6) mean 4 (range 1–8) visits to ED, other hospital, outpatient department before diagnosis	hyponatremia 29/36 severe hyponatremia ( < 125mEq/L) 14/36 transaminases (21/36) (ALT (U/L) 69.5 ± 56.3) anemia 10/36 (haemoglobin (g/L) 117.6 ± 17.9) Misdiagnosis: intestinal obstruction, epilepsy

AHP – acute hepatic porphyria, AIP – acute intermittent porphyria, *m* – male, f – female, ED – emergency department, N/A – not specified, WBC – white blood cell count

The typical presentation of AHP, characterised by neurovisceral symptoms, was consistent across all three studies. Abdominal pain was the most common symptom, affecting nearly all patients (53/59). Although pain intensity was not assessed, one study [105] noted that patients with AHP typically required at least two painkillers (mean 2.6).

Only one study [105] examined patients with undiagnosed AHP who presented to the ED. Their findings suggested that AHP presentations in the ED can be inconclusive and may evolve further over time. Life-threatening progression of respiratory paralysis was reported for 3/59 patients and death occurred in one patient due to delayed diagnosis. [104]

### Patients with familial Mediterranean fever (FMF) in the ED

One study exploring trigger factors in 75 FMF patients presenting to an ED [107] reported the patient characteristics shown in Table 4.

**Table 4:** Characteristics of FMF patients in the ed

	Huseyin et al. 2014
Patients (female, male)	*N* = 75 (f: 41, m: 34)
Age (mean ± SD) per cohort	38.7 ± 12.6 and 33.1 ± 12.1
FMF known	Yes, 7.4–8 (1–24) attacks per year (70/75 patients received treatment with colchicine)
Reason for ED admission	Abdominal pain
family history of FMF	Yes 41/75
trigger factors	Yes 75/75 - Emotional stress (54/75) - Physical activity (40/75) - Menstruation cycle (22/75) - Cold (20/75)

SD – standard deviation, ED – emergency department, FMF – familial Mediterranean fever, m – male, f – female

### Patients with hereditary angioedema (HAE) in the ED

Five retrospective [108, 109, 111–113] and two prospective [25, 110] studies investigated HAE presentation in at least 84 patients presenting to the ED. In two studies [108, 109], HAE only formed a subgroup of patients with unspecified angioedema in the ED. The extracted characteristics are listed in Table 5.

**Table 5:** Characteristics of patients with HAE in the ED

Author, Year	Patients	HAE known	Presentation in ED	Symptoms	Family history of HAE	Trigger factors	Additional findings
Day et al. 2023	n = 11 (f: 10, m: 1)	Yes	Abdominal angioedema, no additional sites affected (36.4%) Swelling above the shoulder (54.5%)	N/A	N/A	Spontaneous: 8/11 Possible trigger reported: 3/11	Time from angioedema onset to presentation was longer in HAE-patients (median time: 24 (IQR 15 - 48) hours).
	Age (median): Study population: 57 (IQR 43 - 67) years HAE-subgroup: N/A						
	Manifestation age: N/A						
Felder et al. 2014	n = 455, HAE-subgroup NA	Yes	Localisation of angioedema per patient visit to the ED: - Periorbital (10%) - Lips (10%) - Tongue (26%) - Face/cheeks (20%) - Pharynx (38%) - Extremities (52%) - multiple localisations (44%)	N/A	N/A	N/A	Significant association between cause and localisation of angioedema: angioedema of the extremities associated with HAE
	Age (mean): All 37.8 (SD 22.8) years HAE-subgroup: N/A						
	Manifestation age: N/A						
Moldovan et al. 2018	n = 3 (f: 2, m: 1) Presentation of 3 patients with fatal laryngeal oedema	Yes 2 No 1	Manifestation of laryngeal oedema with: - Dysphagia, dysphonia and dyspnoea (1/3) - Dysphonia (1/3) - Feeling of a lump in the throat, in the course of dysphagia and dysphonia (1/3)	Frequency of HAE attacks: - weekly (1/3) − 2 ×per month (1/3) - annually (1/3)	Yes (3/3) 3–6 also affected family members	N/A	misdiagnosis led to surgical removal of gall bladder, appendix, ovary Time from onset of laryngeal oedema to asphyxia: 20 min − 11 h Diagnostic latency: 30–49 years
	Age: 42, 52, 59 years						
	Manifestation age: 8, 20, 22 years						
Hirose et al. 2017	n = 3 (f: 2, m: 1)	No	Facial oedema that does not respond to antihistamines or steroids (1/3) Abdominal pain + vomiting (1/3) Out-of-hospital cardiac arrest - onset of facial oedema 20 hours earlier (1/3)	N/A	Yes 2/3 No 1/3	N/A	Routine laboratory: WBC count higher (13 820 [IQR: 10 400–23 200] vs 7750 [IQR: 6167.5–11 057.5]/mL, *p* = 0.04)) Other routine laboratory parameters normal. Imaging: CT- face and neck: laryngeal oedema(1/3) CT-abdomen: intestinales oedema + ascites(1/3) Misdiagnosis: Gastroenteritis, Endometriosis
	Age (median): 52 (IQR 38–85) years						
	Manifestation age: 13, 25, N/A years						
Javaud et al. 2015	n = 29 (f: 19, m: 10) 57 HAE.attacks in 29 patients	Yes	Angioedema localisation: - face (49%) - abdomen (40%) - larynx (32%) - tongue (16%) - extremities (14%) - multiple sites (18/57)	N/A	N/A	Trigger identified for 31/57 attacks: - stress (35%) - trauma (23%) - estrogen (contraception, pregnancy) (23%) - infection (13%) - Discontinuing HAE-Prophylaxis (6%)	management required: - ICU: 6 (21%) - Intubation: 2 (7%) - Tracheotomy: 1
	Age (median): 29 (IQR 26–36) years						
	Manifestation age: N/A						
Pekdemir et al. 2007	n = 3 (f: 3)	Yes 2 N/A. 1	- Shortness of breath, oedema on left arm and rash (since 24 h) (1/3) - Angioedema since 12 h: swelling of the face, especially periorbital; mild abdominal cramps (1/3) - Bilateral oedema of the hand since 12 h	N/A	Yes 1/3 N/A 2/3	Menstruation, infection of the upper airways	Vital signs normal (3/3) Presentation to 2 other medical centres without improvement after treatment with steroids, antihistamines and epinephrine.
	Age: 21, 33, 35 years						
	Manifestation age: 4, 29, N/A						
Songur Kodik et al. 2023	n = 35 (f: 19, m: 16)	Yes and No, not further specified	Most frequent: abdominal pain Localisation of HAE attack: - Abdomen 13 (37.2%) - Uvula 15 (42.9%) - Face 10 (28.6%) - Lips 2 (5.8%) - Trunk/trunk 3 (8.6%)	**GI symptoms:** Abdominal pain 13/35 (37%), Nausea/vomiting 3/35 (8.6%) **Cardiovascular:** Chest pain 1/35 (2.9%), Syncope/presyncope 2/35 (5.7%) **Respiratory:** dyspnoea 7/35 (20%) **Skin:** Facial swelling 11/35 (31.4%), Body swelling 9/35 (25.7%), Urticaria 0/35 **ENT:** Sore throat 9/35 (25.7%) Dysphonia 3/35 (8.6%), Dysphagia 1/35 (2.9%)	Yes 32/35 (91.4%)	Trauma (1/35)	Mean vital signs normal.
	Age (mean): 36.4 (SD 13) years						

N/A – not specified, ICU – intensive care unit, SD – standard deviation, IQR – interquartile range, m – male, f – female, ED – emergency department, WBC – white blood cell count, GI – Gastrointestinal, ENT - Ear, nose and throat, ICU – intensive care unit

The most common presentation of HAE patients in the ED was “swelling above the shoulder” [108], indicating angioedema of the face (lips, periorbital and cheeks), tongue, uvula and/or pharynx. The leading symptoms were abdominal pain and swelling of the face and/or body.

Most of the included studies had a primary study objective other than characterising patients with HAE in the ED.

Swelling of the extremities plus the absence of urticaria should suggest HAE in the ED [109]. HAE patients often had a history of similar previous ED visits and therapy attempts with steroids, epinephrine and antihistamines did not result in any improvement [112].

One study [111] evaluated what went wrong in the ED and proposed lessons learned [111]: misjudgement/insufficient assessment of severity and type of angioedema by the emergency physician and insufficient awareness of rapid HAE progression led to

1. Misdiagnosis and ineffective treatment.
2. Airway not secured and emergency measures not prepared.
3. Delay in providing life-saving treatment resulting in patients’ death.

Therefore, the authors concluded that emergency physicians need to be aware of the possible rapid progression of angioedema in patients with HAE and need to have measures in place to secure the airway and even be prepared for cricothyrotomy.

### Patients with hereditary hemorrhagic telangiectasia (HHT) in the ED

For HHT, a case series [114] describing the initial presentation of three patients with HHT and CNS lesions in the ED was identified. The extracted characteristics are listed in Table 6.

**Table 6:** Characteristics of HHT patients in the ed

	Kuwayama et al. 2003
Patients (female, male)	n = 3 (f: 1, m:2)
Age (years)	23, 32, 62
Age of manifestation	N/A Epistaxis since childhood: 3/3
HHT known	No
ED presentation	Hemiparesis right + loss of consciousness (1/3) Loss of consciousness for a few minutes after drinking alcohol (1/3) Sudden onset and worsening of left arm paresis for 1 day (1/3)
family history of recurrent epistaxis	Yes 2/3 (1/3 N/A) 1^st^ degree family member with HHT – Yes 1/3
Additional findings	Patient 1 (23 years) Normal CT on admission DSA: occlusion of cerebral artery Chest radiography: shadow in left lung Polycythemia (Hemoglobin 17.6 g/dl) Blood coagulation analysis normal Patient 2 (62 years) MRI: ring enhanced lesion temporal lobe Chest radiography: perihilar consolidation laboratory: mild infection Patient 3 (32 years) CT: subcortical hemorrhage MRI: no further findings laboratory: normal Chest-CT/pulmonary arteriography: Pulmonary arteriovenous malformations 3/3

N/A – not specified, DSA – digital subtraction angiography, ED – emergency department, HHT – hereditary haemorrhagic telangiectasia, *m* – male, f – female

All three patients presented with mucocutaneous telangiectasia (nose, tongue, oesophagus, stomach) and reported recurring epistaxis since childhood. Two patients had either a family history of recurrent epistaxis, telangiectasias or HHT diagnosis itself. Pulmonary AVMs were diagnosed in all three patients. The authors concluded that earlier diagnosis of HHT and screening for AVMs could have prevented the occurrence of complications such as ischemic stroke and brain abscess. They stressed the importance of paying attention to patients’ history (nosebleeds since childhood) and their family history in the ED.

### Patients with myasthenia gravis (MG) in the ED

One US publication from 2005 [72] presented a case series of three patients (Table 7). The primary presenting feature in the ED was ptosis.

**Table 7:** Characteristics of patients with mg in the ED

	Smulowitz et al. 2005
Patients (female, male)	n = 3 (f: 3)
Age (years)	48–78
Manifestation age of MG (years)	48, 65, N/A
MG diagnosis known	Yes 1/3 No 2/3
Symptoms	**Ptosis (3/3)** - one sided (2/3) - both sides (1/3) **Diplopia (2/3)** - fluctuating (1/3) - constantly (1/3) **Dyspnoea (1/3)** **Other (1/3)** - Difficulty swallowing (including own saliva) - symmetric mild paresis of extremities
ED presentation	Diplopia (2/3) Increased dyspnea and dysphagia after initial improvement after treatment for pre-existing pneumonia (1/3)
trigger factors	Patient with myasthenic crisis: pneumonia and surgery (1/3) N/A 2/3
Additional findings	MRI (head) und CT (thorax): normal (2/3, 1 N/A) Vital signs: normal (1/3), respiratory rate 20 and SaO2 98% under 2 L oxygen (1/3), (1 N/A) Patient with myasthenic crisis: FVC 1.2 L (normal 4.5–5 L) One patient reported a previous episode of diplopia one month prior to ED presentation.

N/A – not specified, FVC – forced vital capacity

The authors recommended considering MG in all patients exhibiting muscle weakness and myasthenic crisis in those with known MG plus dyspnoea.

Although two publications [119, 120] described patients with myasthenic crisis, the setting and presentation in the ED were unclear. Additionally, four publications [121–124] detailed MG patients with specific key symptoms in the ED and provided guidance for distinguishing them from other differential diagnoses. However, none of these publications characterised the presentation of MG specifically in the ED and therefore could not be included.

### Patients with thrombotic Thrombocytopenic Purpura (TTP) in the ED

Five studies [103, 115–118] characterised 70 adult patients with TTP who presented to the ED (Table 8). Most patients (64/70) presented with their first episode of TTP.

**Table 8:** Characteristics of patients with TTP in the ED

Author, Year	Patients	TTP known	Presentation in ED	Symptoms	Laboratory	Additional findings
Boisriou et al. 2023	n = 40 (with TMA) 29/40 TTP (72.5%) 2/40 HUS (5%) 9/40 other TMA (22.5%) (f: 27 (67.5%), m: 13 (32.5%))	No	**Symptoms of patients with TMA at arrival at ED:** **Neurological 10 (25%)** Headache 4 (10%) Aphasia 6 (15%) Confusion 4 (10%) Paraesthesia 2 (5%) Ataxia 1 Paralysis 1 Seizure 2 Loss of vision 1 **GI symptoms 12 (30%)** Vomiting 4 (10%) Abdominal pain 9 (22.5%) **Cardiovascular 4 (10%)** Dyspnoea 2 (5%) Hypertension 3 (7.5%) Chest pain 1 **Haemorrhage 20 (50%)** Cutaneous and mucosal 17 (42.5%) **Acute renal failure 9 (22.5%)** **Other** Jaundice/pallor 7 (17.5%)	**Symptoms of patients with TMA prior to arrival at ED:** **Neurological 20 (50%)** Headache 13 (32.5%) Aphasia 6 (15%) Confusion 4 (10%) Paraesthesia 3 (7.5%) Ataxia 2 (5%) Paralysis 1 Seizure 1 Loss of vision 1 **GI symptoms 17 (42.5%)** Vomiting 11 (27.5%) Diarrhoea 8 (20%) Abdominal pain 7 (17.5%) **Cardiovascular 11 (27.5%)** Dyspnoea 6 (15%) Hypertension 4 (10%) Chest pain 1 **Haemorrhage 16 (40%)** Cutaneous and mucosal 11 (27.5%), Gynaecological 4 (10%), GI 2 (5%), Urological 2 (5%) **Urological 9 (22.5%)** Brown urine 8 (20%), Anuria 1 **Other** Fatigue 14 (35%), Fever 13 (32.5%), Yellow colouration 1, Diffuse pain 3 (7.5%)	Median platelet count (/mm3) 17,000 (IQR 10,000– 39,250) Median haemoglobin (g/dL) 9.5 (IQR 7.4 - 10.5) Schistocytes present 28/34 (82.4%) (6 N/A) Elevated hsTroponin I ( > 14 ng/L) 15/24 (62.5%) (16 N/A)	Pregnancy/Postpartum 1/40 Duration of symptoms before arrival in the emergency department: median 4 days (IQR 3–7) Death 4/40 Misdiagnosis: Infection, immune thrombocytopenia, autoimmune haemolytic anaemia, stroke In 16/40 (40%) of patients, the diagnosis of TMA, TTP or HUS was not mentioned, despite the presence of thrombocytopenia, anaemia and schistocytes in 6 of these cases (37.5%)
	Age (Median): 44 (IQR 28–57) years					
Li et al. 2021	n = 19 (f:10, m:9)	No	**Impaired consciousness** (9/19) and: - Purpura (3/19) - Skin ecchymoses (1/19) - Fatigue, fever (1/19) - Dizziness, fatigue, subcutaneous ecchymoses (1/19) **Vomiting** (3/19) and: - Palpitations (1/19) - Ecchymoses on extremities, fever (1/19) - Nausea, jaundice (1/19) **Haemorrhage** (2/19): - bleeding wounds (1/19) - Blood in sputum and stool, headache (1/19) **Other** (5/19): - Dizziness, fatigue, slurred speech (1/19) - Skin ecchymoses, difficulty opening mouth (1/19) - Chest tightness and sore throat (1/19) - Oedema, joint pain, cough, sputum, shortness of breath (1/19) - Loss of appetite (1/19)	**Neurological symptoms** 11 (58%) Symptoms of thrombocytopenia* 8 (42%) **Fever** 2 (11%) **Renal function impairment** 6 (32%) **Classical pentad** 4 (21%) *Symptoms of thrombocytopenia: petechiae, haematomas, melena, haematuria, haematemesis	Platelet count: median 12 × 10^9/L (3–99 ×10^9/L) Haemoglobin: 71 g/L (44–108 g/L) Schistocytes (9/19): 5.7% (0.3–40%)	Discussed trigger: Idiopathic 10/19 (53%) Surgery 1/19 Pregnancy/Postpartum: 2/19 Connective tissue disease: 6/19 Median time between symptom onset and diagnosis: 7 days (2 days to 3 month) Death 4/19
	Age (median): 56 (range 19–75) years					
Noel et al. 2013	n = 6 (f:4, m:2)	N/A	N/A	Neurological symptoms 4/6 (66.6%) Acute renal failure 2/6 (33.3%)	Haemoglobin (g/l) 69 [48–94, 96–98, 100–111] Schistocytes, n (%) 6 [101] Platelet count (10^9/L) 12.5 (8–41) Median (min-max)	Death 0/6
	Age (median): 35 (range 23 - 74) years					
Pieralli et al. 2011	n = 12 (f:58%, m:42%)	No	Fluctuating neurological symptoms 9/12 (83%) Fever 9/12 (83%) Weakness 10/12 (91%) Renal failure 4/12 (36%) Purpura 2/12 Haematuria 2/12 Abdominal pain 1/12	Most frequent: fluctuating neurological symptoms (e.g. focal deficits, coma, seizure) and fever	Platelet count 24,300 (9000 - 63,000) (cell/L) Haemoglobin (g/dL) 7.2 (4.5–11.7) Mean schistocyte count per field of view 3 (2 - 6) Creatinine (mg/dL) 1.5 (0.8–2.1) (mean)	Discussed trigger/etiology: Pregnancy/Postpartum: 1/12 Ticlopidin 2/12 Unknown 9/12 Timed between symptom onset and diagnosis (mean): 6 days (range 3–36) Death 5/12 (42%) Misdiagnosis: Depression, anxiety, thyroid disease (misdiagnoses, especially in younger patients)
	Age (mean): 59 (range 37–85) years Women were significantly younger than men (50.5 vs. 67 years)					
Stella et al. 2009	n = 12, ED - subgroup = 4, all female and pregnant	No 11/12 Yes 1/12 Diagnosis not known in ED-subgroup.	Anxiety (1/4) Nausea, vomiting (1/4) Loss of consciousness (1/4) Loss of consciousness, confused (1/4) 2/4 were discharged home 2–3 times before being hospitalised	Nausea, vomiting, abdominal pain and changes in the state of consciousness were the most common symptoms. Fever: 0/4.	Platelet count 3–13 ×(10^3^/mm^3^) Hematocrite: 20.6 - 24.2% (range)	Duration between symptom onset and diagnosis 1–6 days Maternal Death: 0/4 (ED presentation), 3/12 (total, *n* = 12) Fetal Death: 1/4, 2/12 Misdiagnosis: Panic attack, domestic violence, gastroenteritis
	Age (range): 20–40 years Gestational age: 12, 21, 24, 30 weeks					

ED – emergency department, HUS - hemolytic uremic syndrome, TMA – thrombotic microangiopathy, TTP – thrombotic thrombocytopenic purpura, f – female, m – male, GI – Gastrointestinal, N/A – not specified

The most common presentations in the ED were neurological and/or gastrointestinal symptoms and weakness; in one patient, anxiety was noted as the ED presentation. Misdiagnosis was reported specifically in younger patients [118].

The median duration of symptoms before presentation to the ED ranged from 4 to 7 days.

One study [103] specifically investigated TTP in pregnant patients. Among the four included patients, two were sent home two to three times from the ED before hospitalisation. All four women survived; however, one of the four unborn children died. The overall mortality rate reported in all the studies ranged from 0% to 42% [117].

### Common features of patients with RD in the ED

On the basis of the findings for each individual investigated RD, common features identified at the time of presentation to the ED are shown in Table 9. In case a common feature was not applicable for all patients evaluated for an RD, it was distinguished between described for at least two third of patients included in the review (= ✓) and described for less than two third of patients (= (✓)). To assess the robustness of results, a comparative overview of study design, patient numbers and extracted outcomes across all RD is provided in Table 2.

**Table 9:** Characteristics of RD presentation in the ED

RD (No. of patients included in this review)	Age < 65 years	Organ systems affected	Recurrent episodes	Vital signs	Routine laboratory	Imaging	Trigger factor present	Family history
AHP (n = 59)	✓	Gastrointestinal, Neurologic, Urologic, Cardiovascular	mean 4 (range 1-8) visits to ED, other hospital or outpatient department before diagnosis † cyclic attacks †	Hypertensive 7/49 Tachycardic 10/36 N/A: 10/59	Hyponatremia▽	Abdominal ultrasound, head-CT/MRI normal †	✓*	(✓)*
FMF † (n = 75)	✓	—	✓	—	—	—	✓	✓
HAE (n >84)	✓°	Gastrointestinal, Cardiovascular, Respiratory, Skin, ENT †	Weekly (1/3), two per month (1/3), annually (1/3) †	Normal*	WBC↑ †	CT face and neck: laryngeal oedema (1/3), CT abdomen: intestinal oedema + ascites (1/3) †	(✓)*	✓*
HHT † (n = 3)	✓	Neurological, ENT, Skin, Respiratory	✓	—	Polycythaemia, mild infection	Chest radiography: shadow in left lung/perihilar consolidation Chest-CT/pulmonary arteriography: Pulmonary arteriovenous malformations CNS lesion in DSA, MRI, CT	—	✓
MG † (n = 3)	(✓)	Ophthalmologic, Neurologic, ENT, Respiratory	(✓)	Normal Patient with myasthenic crisis had normal vital signs except for a respiratory rate of 20 and O_2_ saturation of 98% under 2L of oxygen.	—	Normal MRI (head) und CT (thorax)	(✓)	—
TTP (n = 70)	✓°	Neurologic, Gastrointestinal, Cardiovascular, Urologic, Gynaecologic, ENT, Orthopaedic, Skin, Psychiatric, General (Fatigue, Fever)	2/4 patients had been sent home 2–3 times before being hospitalized*	—	Thrombocytopenia ▽ Schistocytes ▽ Anaemia ▽ Signs of end organ damage (Troponin ↑, Creatinin ↑)▽	—	(✓)*	—

✓= described for at least two third of patients included in the review

(✓)= described for less than two third of patients

(—)= not assessed

* = not assessed by all studies

† = assessed by only one study

° = based on mean and median

▽ = disease specific characteristic

WBC — white blood cell count, DSA — digital subtraction angiography

The listed organ systems were not affected in all patients at the same time. Basic imaging (ultrasound, CT, MRI, chest radiography) was normal (AHP, MG), showed nonspecific findings (oedema or ascites in HAE, shadow or consolidation in chest radiography in HHT) or was not assessed (FMF, TTP). Normal or nonspecific findings (including disease specific findings e.g. hyponatremia in AHP) in standard imaging and laboratory results were therefore a common feature among patients with AHP, HAE, MG and HHT.

Two additional characteristics were observed in patients with RD who presented to the ED. The first is the presence of trigger factors reported for patients with AHP, FMF, HAE, MG and TTP and the second is a family history of similar symptoms or even RD itself (AHP, FMF, HAE, HHT).

## Discussion

In this systematised review of primary data, 18 studies were assessed for common features among patients with rare but treatable diseases presenting to the ED. The studies were all published after 2003. The ED presentation included both diagnosed and undiagnosed RD patients. Some studies did not provide information on whether the diagnosis was already established or not.

The common features identified in this review were patient age < 65 years; symptoms affecting multiple organ systems contributing to a nonspecific presentation; recurrence of symptoms reflected by previous presentation to other health institutions with similar symptoms or a history of similar previous episodes; the presence of trigger factors; nonindicative or normal findings in routine laboratory, imaging and vital signs; and a positive family history.

Deviations from these common features can occur especially in vital signs, laboratory tests, imaging and family history. For example disease specific and therefore non-common features were hyponatremia, tachycardia and hypertension in AHP patients, signs of oedema on CT scans of HAE patients and severe thrombocytopenia and haemolytic anaemia in TTP patients and a negative family history in patients with MG. Nevertheless, a positive family history was a common feature among other RD and can serve as a strong red flag indicating an RD in the ED if present. RD represents a highly heterogeneous group of conditions that can manifest in very individual ways, making it challenging to derive common features and red flags across them. This list should not be viewed as a checklist to rule out an RD in the ED. Instead, it serves as an initial attempt to describe patients with RD in the ED, providing a starting point for further research aimed at enhancing care for RD patients, potentially by utilising these common features.

Previous studies have described general characteristics of RD patients outside the ED setting. A recent study derived seven biopsychosocial red flags in patients with RD from reports of RD patient groups [125]. In line with some findings in our review, they also identified the involvement of multiple organ systems (at least three) and a positive family history as sign of a genetic inheritance as common features. They also reported a continuance of presentation from child- to adulthood, which was also mentioned in the studies included in this review on HHT (epistaxis since childhood) and HAE (recurrent angioedema since childhood), referrals to multiple specialists, which were reported in the included studies in our review as multiple health care or ED visits described for AHP, HAE and TTP patients and misdiagnosis. In addition to our characteristics, they reported difficulties in school (absence, problems participating in physical activities, bullying and social isolation) and prolonged unexplained symptoms.

Another study defined characteristics for patients that should prompt further testing to exclude an RD [126]. These consisted of physical changes without sufficient explanation affecting multiple organ systems that began in childhood or adolescence and may similarly be encountered in the patient’s family, with no improvement despite therapy, a duration of complaints of more than six months to one year without a diagnosis and avoidance of generally welcomed activities. All three sets of potential warning signs of the underlying RD are summarised in Table 10.

**Table 10:** Potential red flags of patients with RD

Potential red flags derived from this review	**Grigull 2021** [126]	Al-Attar et al. 2024 [125]
- Multiple organ systems affected, nonspecific presentation - Recurrent symptoms, previous presentation to health institutions - Patient age < 65 years - Positive family history - Nonspecific or normal findings in: routine laboratory, standard imaging (chest radiography, CT, MRI, ultrasound), vital signs - Trigger factor present	- Several organ systems are affected - > 6–12 months of complaints and search for a diagnosis (in adults) - Specific features go back to childhood/adolescence, possibly also similar features in the family environment - Irritating physical changes without explanation - No improvement despite adequate therapy - Deliberate avoidance of certain, generally welcome activities (e.g. holidays, meetings with friends)	- Multi-system involvement (3 or more) - Multiple specialist referrals - Presentation in both childhood and adulthood - Genetic inheritance pattern - Delayed diagnosis (no diagnosis after one year of seeking medical help) - Misdiagnosis - Difficulties at school e.g. especially absences, difficulty participating in physical education and bullying/social isolation

The identified common features of RD patients in the ED and the validity for RD outside of the predefined set of RD need to be assessed in further studies, particularly with respect to their applicability beyond the predefined set of RD.

Several studies have emphasised the need for immediate diagnosis and treatment of RD in the ED to prevent possible fatal outcomes [25, 104, 111]. In the high-pressure setting of the ED, where time and resources are limited and the primary focus is on stabilising immediate life-threatening conditions, it is crucial to develop criteria that help patients who may benefit from additional diagnostic workup. The identified common features may serve as red flags for RD in the ED, but their effectiveness in this context needs further careful evaluation. Integrating such red flags into triage protocols and decision support systems may strengthen the ability of ED teams to recognise and manage patients with RD. Beyond individual patient care, the implementation of such a system could enhance the overall awareness of RD among emergency staff.

There are several limitations to this review and the thereby identified common features. First, this was a systematised and not fully systematic literature review, mainly performed by a single reviewer. Although the whole search process based on methodological standards (JBI Manual PRISMA), the search strategy was non-exhaustive. While a comprehensive search of additional databases (done exemplarily for AHP) did not yield further eligible studies, the risk of missing relevant studies cannot be fully ruled out. The languages of the included studies were limited to English and German, -although only *n* = 7 studies were ruled out due to language reasons, a language bias remains possible.

The included studies were mostly retrospective chart reviews considering small patient cohorts. Only a few prospective cohort studies could be identified. This reliance on mainly retrospective data may introduce selection bias, as for these studies available medical records may not represent the entire patient population. Excluding case reports may also have led to the omission of valuable information, particularly for RD with limited published research. However, since the review aimed to identify broader trends relevant to larger patient populations, case reports were considered not to contribute significantly to the overall understanding, as they describe specific isolated cases. Patient characteristics were reported very heterogeneously throughout the studies, only 33% (*n* = 6) were designed to characterise patients with RD in the ED. Such limited data may lead to inconsistencies in disease classification and symptom interpretation. The limited number of studies for specific RD (e.g., only one case series each for MG and HHT and none for PNH and FD) increases the risk of misclassification due to insufficient data. Studies were not limited to patients presenting to the ED with undiagnosed RD, which may bias the findings of symptoms and presenting features in the ED in terms of potential overestimation of diagnostic clarity in the ED.

Taken together, these limitations highlight the need for caution when interpreting the results and emphasise the importance of further research. Nevertheless, as mentioned before methodological specifications (JBI Manual, PRISMA) informed the whole review process to ensure the scientific robustness of the results.

Further research is necessary to identify ED specific features of RD that could support risk stratification to identify patients with RD in the ED. To the best of our knowledge, this is the first literature review exploring the common features of patients with RD specifically in the ED context.

## Conclusions

Information available on RD in the emergency care setting remain limited and descriptions of RD presentation in the ED are scarce. This review proposes some common features among patients with RD in the ED that could be used to develop risk stratification algorithms and early warning systems. In practice, these findings could be integrated into structured triage checklists or serve as input for clinical decision support tools including AI based approaches, helping emergency physicians to recognise early signs of RD and initiate prompt treatment, potentially improving patient outcomes and reducing the overall burden on healthcare resources. Further studies should focus on prospectively enrolling patients in the specific ED setting and thus testing and validating these identified common features to determine their applicability and effectiveness in clinical practice. As emphasised before, this is a first approach to identify ED specific findings in patients with RD. We were only able to explore a small and exemplary group of RD, but with rising awareness of RD in the emergency care setting, there will be more diagnoses and therefore more usable data. It is important to address RD in the ED - not least since they are individually rare but collectively common.

## Electronic Supplementary Material

Below is the link to the electronic supplementary material.

Supplementary Material 1

Supplementary Material 2

## Data Availability

All data supporting the findings of this review are available in this paper and its supplementary materials.

## Abbreviations

AHP: Acute hepatic porphyria
AVM: Arteriovenous malformation
ED: Emergency department
FD: Fabry disease
FMF: Familial Mediterranean fever
HAE: Hereditary angioedema
HHT: Hereditary hemorrhagic telangiectasia
MG: Myasthenia gravis
PNH: Paroxysmal nocturnal haemoglobinuria
RD: Rare disease
TTP: Thrombotic thrombocytopenic purpura

## References

[1] What is EM? European Society for Emergency Medicine. https://eusem.org/about-us/emergency-medicine/what-is-em. Accessed 18 March 2025.

[2] Abozaid GM, Kerr K, McKnight A, Al-Omar HA. Criteria to define rare diseases and orphan drugs: a systematic review protocol. BMJ Open. 2022;12(7):e062126. 35906057 10.1136/bmjopen-2022-062126 PMC9345065

[3] Official Journal of the European Communities. Commission regulation (ec) No 847/2000. 2000. https://eur-lex.europa.eu/resource.html?uri=cellar:208111e4-414e-4da5-94c1-852f1c74f351.0008.02/DOC_1%26format=PDF. Accessed 17 April 2025.

[4] Office of the Commissioner. Orphan Drug Act - relevant Excerpts. U.S. Food and Drug administration. 2013. https://www.fda.gov/industry/designating-orphan-product-drugs-and-biological-products/orphan-drug-act-relevant-excerpts. Accessed 12 December 2024.

[5] Richter T, Nestler-Parr S, Babela R, Khan ZM, Tesoro T, Molsen E, et al. Rare disease terminology and definitions-A systematic global review: report of the ispor rare disease special interest group. Value Health. 2015;18(6):906–14. 26409619 10.1016/j.jval.2015.05.008

[6] Haendel M, Vasilevsky N, Unni D, Bologa C, Harris N, Rehm H, et al. How many rare diseases are there? Nat Rev Drug Discov. 2020;19(2):77–78. 32020066 10.1038/d41573-019-00180-y PMC7771654

[7] European Commission. Rare diseases. https://health.ec.europa.eu/rare-diseases-and-european-reference-networks/rare-diseases_en. Accessed 17 April 2025.

[8] Nguengang Wakap S, Lambert DM, Olry A, Rodwell C, Gueydan C, Lanneau V, et al. Estimating cumulative point prevalence of rare diseases: analysis of the Orphanet database. Eur J Hum Genet. 2020;28(2):165–73. 31527858 10.1038/s41431-019-0508-0 PMC6974615

[9] CDC/National Center for Health Statistics. Emergency Department Visits. 2024. https://www.cdc.gov/nchs/fastats/emergency-department.htm. Accessed 17 April 2025.

[10] Reform der Notfall- und Akutversorgung in Deutschland. Integrierte Notfallzentren und Integrierte Leitstellen [Reform of emergency and acute care in Germany. Integrated emergency centres and integrated control centres]. 2023. https://www.bundesgesundheitsministerium.de/fileadmin/Dateien/3_Downloads/K/Krankenhausreform/Vierte_Stellungnahme_Regierungskommission_Notfall_ILS_und_INZ.pdf. Accessed 17 April 2025.

[11] Statista Research Department. Number of visits registered by the emergency services in France from 2017 to 2022 2023. https://www.statista.com/statistics/1369429/number-of-emergency-visits-in-france/. Accessed 17 April 2025.

[12] Adachi T, El-Hattab AW, Jain R, Nogales Crespo KA, Quirland Lazo CI, Scarpa M, et al. Enhancing equitable access to rare disease diagnosis and treatment around the world: a review of evidence, policies, and challenges. Int J Environ Res Public Health. 2023;20(6) 10.3390/ijerph20064732 PMC10049067 36981643

[13] Kole A, Faurisson F. Rare diseases social epidemiology: analysis of inequalities. Adv Exp Med Biol. 2010;686:223–50. 20824449 10.1007/978-90-481-9485-8_14

[14] Faye F, Crocione C, Anido de Pena R, Bellagambi S, Escati Penaloza L, Hunter A, et al. Time to diagnosis and determinants of diagnostic delays of people living with a rare disease: results of a rare barometer retrospective patient survey. Eur J Hum Genet. 2024. 10.1038/s41431-024-01604-z PMC11369105 38755315

[15] Mazzucato M, Visona Dalla Pozza L, Manea S, Minichiello C, Facchin P. A population-based registry as a source of health indicators for rare diseases: the ten-year experience of the Veneto Region’s rare diseases registry. Orphanet J Rare Dis. 2014;9:37. 24646171 10.1186/1750-1172-9-37 PMC4000007

[16] Mazzucato M, Visona Dalla Pozza L, Minichiello C, Toto E, Vianello A, Facchin P. Estimating mortality in rare diseases using a population-based registry, 2002 through 2019. Orphanet J Rare Dis. 2023;18(1):362. 37978388 10.1186/s13023-023-02944-7 PMC10655462

[17] Navarrete-Opazo AA, Singh M, Tisdale A, Cutillo CM, Garrison SR. Can you hear us now? The impact of health-care utilization by rare disease patients in the United States. Genet Med. 2021;23(11):2194–201. 34183788 10.1038/s41436-021-01241-7 PMC8553605

[18] Blazsik RM, Beeler PE, Tarcak K, Cheetham M, von Wyl V, Dressel H. Impact of single and combined rare diseases on adult inpatient outcomes: a retrospective, cross-sectional study of a large inpatient population. Orphanet J Rare Dis. 2021;16(1):105. 33639989 10.1186/s13023-021-01737-0 PMC7913458

[19] Isono M, Kokado M, Kato K. Why does it take so long for rare disease patients to get an accurate diagnosis?-A qualitative investigation of patient experiences of hereditary angioedema. PLoS One. 2022;17(3):e0265847. 35303740 10.1371/journal.pone.0265847 PMC8932585

[20] Zhou L, Xu J, Yang J. Poor education and urgent information need for emergency physicians about rare diseases in China. Orphanet J Rare Dis. 2022;17(1):211. 35619153 10.1186/s13023-022-02354-1 PMC9137093

[21] Faucounneau V, Rath A. Emergency guidelines and emergency cards. Orphanet J Rare Dis. 2014;9(1):O15.

[22] Betschel S, Avilla E, Kanani A, Kastner M, Keith P, Binkley K, et al. Development of the hereditary angioedema rapid triage tool. J Allergy Clin Immunol Pract. 2020;8(1):310–7 e3. 31238160 10.1016/j.jaip.2019.05.056

[23] Solares I, Heredia-Mena C, Castelbon FJ, Jerico D, Cordoba KM, Fontanellas A, et al. Diagnosis and management of inborn errors of metabolism in adult patients in the emergency department. Diagn (Basel). 2021;11(11) 10.3390/diagnostics11112148 PMC8621113 34829496

[24] Masatlioglu S, Dulundu E, Gogus F, Hatemi G, Ozdogan H. The frequency of familial Mediterranean fever in an emergency unit. Clin Exp Rheumatol. 2011;29(4):S44–6. 21968235

[25] Hirose T, Kimbara F, Shinozaki M, Mizushima Y, Yamamoto H, Kishi M, et al. Screening for hereditary angioedema (hae) at 13 emergency centers in Osaka, Japan: a prospective observational study. Med (Baltim). 2017;96(6):e6109. 10.1097/MD.0000000000006109 PMC5313030 28178173

[26] Diehl-Wiesenecker E, Blaschke S, Wohmann N, Kubisch I, Stauch T, Mainert M, et al. Detect acute porphyrias in emergency departments (DePored) - a pilot study. Orphanet J Rare Dis. 2023;18(1):146. 37308920 10.1186/s13023-023-02768-5 PMC10258746

[27] Chirmule N, Feng H, Cyril E, Ghalsasi VV, Choudhury MC. Orphan drug development: challenges, regulation, and success stories. J Biosci. 2024;49. 38383975

[28] Vandeborne L, van Overbeeke E, Dooms M, De Beleyr B, Huys I. Information needs of physicians regarding the diagnosis of rare diseases: a questionnaire-based study in Belgium. Orphanet J Rare Dis. 2019;14(1):99. 31054581 10.1186/s13023-019-1075-8 PMC6500578

[29] Elder G, Harper P, Badminton M, Sandberg S, Deybach JC. The incidence of inherited porphyrias in Europe. J Inherit Metab Dis. 2013;36(5):849–57. 23114748 10.1007/s10545-012-9544-4

[30] Bonkovsky HL, Maddukuri VC, Yazici C, Anderson KE, Bissell DM, Bloomer JR, et al. Acute porphyrias in the Usa: features of 108 subjects from porphyrias consortium. Am J Med. 2014;127(12):1233–41. 25016127 10.1016/j.amjmed.2014.06.036 PMC4563803

[31] Andersson C, Innala E, Backstrom T. Acute intermittent porphyria in women: clinical expression, use and experience of exogenous sex hormones. A population-based study in northern Sweden. J Intern Med. 2003;254(2):176–83. 12859699 10.1046/j.1365-2796.2003.01172.x

[32] Diehl-Wiesenecker E, Somasundaram R. 409 Die Porphyrien [The porphyrias]. In: Suttorp N, Möckel M, Siegmund B, Dietel M, editors. Harrisons Innere Medizin [Harrison’s principles of internal medicine, German edition]. 20th ed. ABW Verlag; 2020. p. 3701–14.

[33] Stolzel U, Doss MO, Schuppan D. Clinical Guide and update on porphyrias. Gastroenterology. 2019;157(2):365–81 e4. 31085196 10.1053/j.gastro.2019.04.050

[34] Ventura P, Bonkovsky HL, Gouya L, Aguilera-Peiro P, Montgomery Bissell D, Stein PE, et al. Efficacy and safety of givosiran for acute hepatic porphyria: 24-month interim analysis of the randomized phase 3 envision study. Liver Int. 2022;42(1):161–72. 34717041 10.1111/liv.15090 PMC9299194

[35] Yasuda M, Keel S, Balwani M. Rna interference therapy in acute hepatic porphyrias. Blood. 2023;142(19):1589–99. 37027823 10.1182/blood.2022018662 PMC10656724

[36] Arends M, Wanner C, Hughes D, Mehta A, Oder D, Watkinson OT, et al. Characterization of classical and nonclassical fabry disease: a multicenter study. J Am Soc Nephrol. 2017;28(5):1631–41. 27979989 10.1681/ASN.2016090964 PMC5407735

[37] Schiffmann R, Warnock DG, Banikazemi M, Bultas J, Linthorst GE, Packman S, et al. Fabry disease: progression of nephropathy, and prevalence of cardiac and cerebrovascular events before enzyme replacement therapy. Nephrol Dial Transpl. 2009;24(7):2102–11. 10.1093/ndt/gfp031 PMC2698092 19218538

[38] Eng CM, Fletcher J, Wilcox WR, Waldek S, Scott CR, Sillence DO, et al. Fabry disease: baseline medical characteristics of a cohort of 1765 males and females in the fabry registry. J Inherit Metab Dis. 2007;30(2):184–92. 17347915 10.1007/s10545-007-0521-2

[39] Meikle PJ, Hopwood JJ, Clague AE, Carey WF. Prevalence of lysosomal storage disorders. Jama. 1999;281(3):249–54. 9918480 10.1001/jama.281.3.249

[40] Poorthuis BJ, Wevers RA, Kleijer WJ, Groener JE, de Jong JG, van Weely S, et al. The frequency of lysosomal storage diseases in the Netherlands. Hum Genet. 1999;105(1–2):151–56. 10480370 10.1007/s004399900075

[41] Gambardella J, Riccio E, Bianco A, Fiordelisi A, Cerasuolo FA, Buonaiuto A, et al. Fatigue as hallmark of fabry disease: role of bioenergetic alterations. Front. Cardiovasc. Med. 2024;11:1341590. 38327490 10.3389/fcvm.2024.1341590 PMC10847249

[42] Üçeyler N, Abicht A, Beck M, Brand E, Jünemann A, Vom Dah S. Diagnose und Therapie des Morbus Fabry, S1-Leitlinie. [Diagnosis and treatment of fabry disease, S1 guideline]. Deutsche Gesellschaft für Neurologie Leitlinien für Diagnostik und Therapie in der Neurologie. 2022. https://register.awmf.org/assets/guidelines/030-134l_S1_Diagnostik-Therapie-Morbus-Fabry_2023-02.pdf. Accessed 1 June 2024.

[43] Belmatoug N, Bagou G. Orphanet emergencies. Maladie de Fabry [Fabry disease]. 2011. https://www.orpha.net/pdfs/data/patho/Pro/fr/Urgences_Fabry.pdf. Accessed 19 April 2025.

[44] Ben-Chetrit E, Levy M. Familial Mediterranean fever. Lancet. 1998;351(9103):659–64. 9500348 10.1016/S0140-6736(97)09408-7

[45] Aydin O, Egeli BH, Ozdogan H, Ugurlu S. Late-onset familial mediterranean fever: single-center experience and literature review. Intern Emerg Med. 2022. 10.1007/s11739-021-02912-8 35061158

[46] Sohar E, Gafni J, Pras M, Heller H. Familial Mediterranean fever. A survey of 470 cases and review of the literature. Am J Med. 1967;43(2):227–53. 5340644 10.1016/0002-9343(67)90167-2

[47] Ben-Chetrit E, Touitou I. Familial mediterranean fever in the world. Arthritis Rheum. 2009;61(10):1447–53. 19790133 10.1002/art.24458

[48] Rowczenio DM, Iancu DS, Trojer H, Gilbertson JA, Gillmore JD, Wechalekar AD, et al. Autosomal dominant familial Mediterranean fever in Northern European caucasians associated with deletion of p.M694 residue-a case series and genetic exploration. Rheumatol (Oxford). 2017;56(2):209–13. 10.1093/rheumatology/kew058 27150194

[49] Zadeh N, Getzug T, Grody WW. Diagnosis and management of familial Mediterranean fever: integrating medical genetics in a dedicated interdisciplinary clinic. Genet Med. 2011;13(3):263–69. 21317656 10.1097/GIM.0b013e31820e27b1

[50] Lancieri M, Bustaffa M, Palmeri S, Prigione I, Penco F, Papa R, et al. An update on familial Mediterranean fever. Int J Mol Sci. 2023;24(11) 10.3390/ijms24119584 PMC10253709 37298536

[51] Ozturk MA, Kanbay M, Kasapoglu B, Onat AM, Guz G, Furst DE, et al. Therapeutic approach to familial Mediterranean fever: a review update. Clin Exp Rheumatol. 2011;29(4 Suppl 67):S77–86. 21968242

[52] Kharouf F, Tsemach-Toren T, Ben-Chetrit E. IL-1 inhibition in familial Mediterranean fever: clinical outcomes and expectations. Clin Exp Rheumatol. 2022;40(8):1567–74. 36062765 10.55563/clinexprheumatol/obb2ds

[53] Georgin-Lavialle S, Savey L, Cuisset L, Boursier G, Boffa JJ, Delplanque M, et al. French protocol for the diagnosis and management of familial Mediterranean fever. Rev Med Interne. 2023;44(11):602–16. 37903671 10.1016/j.revmed.2023.10.441

[54] Ozen S, Demirkaya E, Erer B, Livneh A, Ben-Chetrit E, Giancane G, et al. EULAR recommendations for the management of familial Mediterranean fever. Ann Rheum Dis. 2016;75(4):644–51. 26802180 10.1136/annrheumdis-2015-208690

[55] Aygoren-Pursun E, Magerl M, Maetzel A, Maurer M. Epidemiology of bradykinin-mediated angioedema: a systematic investigation of epidemiological studies. Orphanet J Rare Dis. 2018;13(1):73. 29728119 10.1186/s13023-018-0815-5 PMC5935942

[56] Bork K, Meng G, Staubach P, Hardt J. Hereditary angioedema: new findings concerning symptoms, affected organs, and course. Am J Med. 2006;119(3):267–74. 16490473 10.1016/j.amjmed.2005.09.064

[57] Maurer M, Magerl M, Betschel S, Aberer W, Ansotegui IJ, Aygoren-Pursun E, et al. The international WAO/EAACI guideline for the management of hereditary angioedema-the 2021 revision and update. Allergy. 2022;77(7):1961–90. 35006617 10.1111/all.15214

[58] Plauchu H, de Chadarevian Jp, Bideau A, Robert JM. Age-related clinical profile of hereditary hemorrhagic telangiectasia in an epidemiologically recruited population. Am J Med Genet. 1989;32(3):291–97. 2729347 10.1002/ajmg.1320320302

[59] Serra MM, Papi M, Serrano C. Prevalence of hereditary hemorrhagic telangiectasia in a medical care program organization in Buenos Aires, Argentina. Medicina (B Aires). 2024;84(2):221–26. 38683506

[60] Shovlin CL, Guttmacher AE, Buscarini E, Faughnan ME, Hyland RH, Westermann CJ, et al. Diagnostic criteria for hereditary hemorrhagic telangiectasia (rendu-osler-weber syndrome). Am J Med Genet. 2000;91(1):66–67. 10751092 10.1002/(sici)1096-8628(20000306)91:1<66::aid-ajmg12>3.0.co;2-p

[61] Dupuis-Girod S, Cottin V, Shovlin CL. The lung in hereditary hemorrhagic telangiectasia. Respiration. 2017;94(4):315–30. 28850955 10.1159/000479632

[62] Shovlin CL, Buscarini E, Sabba C, Mager HJ, Kjeldsen AD, Pagella F, et al. The European rare disease network for HHT frameworks for management of hereditary haemorrhagic telangiectasia in general and speciality care. Eur J Med Genet. 2022;65(1):104370. 34737116 10.1016/j.ejmg.2021.104370

[63] Faughnan ME, Palda VA, Garcia-Tsao G, Geisthoff UW, McDonald J, Proctor DD, et al. International guidelines for the diagnosis and management of hereditary haemorrhagic telangiectasia. J Med Genet. 2011;48(2):73–87. 19553198 10.1136/jmg.2009.069013

[64] Faughnan ME, Mager JJ, Hetts SW, Palda VA, Lang-Robertson K, Buscarini E, et al. Second international guidelines for the diagnosis and management of hereditary hemorrhagic telangiectasia. Ann Intern Med. 2020;173(12):989–1001. 32894695 10.7326/M20-1443

[65] Carr AS, Cardwell CR, Po M, McConville J. A systematic review of population based epidemiological studies in Myasthenia Gravis. BMC Neurol. 2010;10:46. 20565885 10.1186/1471-2377-10-46 PMC2905354

[66] Deenen JC, Horlings CG, Verschuuren JJ, Verbeek AL, van Engelen BG. The epidemiology of neuromuscular disorders: a comprehensive overview of the literature. J Neuromuscul Dis. 2015;2(1):73–85. 28198707

[67] Vissing J, Atula S, Savolainen M, Mehtala J, Mehkri L, Olesen TB, et al. Epidemiology of myasthenia gravis in Denmark, Finland and Sweden: a population-based observational study. J Neurol Neurosurg Psychiatry. 2024. 10.1136/jnnp-2023-333097 PMC11420710 38538059

[68] Keovilayhong S, Mulliez A, Feral L, Chenaf C, Clavelou P, Moisset X, et al. Epidemiology of myasthenia gravis in France: incidence, prevalence, and comorbidities based on national healthcare insurance claims data. Rev Neurol (paris). 2024;180(5):451–58. 38582663 10.1016/j.neurol.2024.02.392

[69] Punga AR, Maddison P, Heckmann JM, Guptill JT, Evoli A. Epidemiology, diagnostics, and biomarkers of autoimmune neuromuscular junction disorders. The Lancet Neurol. 2022;21(2):176–88. 35065040 10.1016/S1474-4422(21)00297-0

[70] Li F, Hotter B, Swierzy M, Ismail M, Meisel A, Ruckert JC. Generalization after ocular onset in myasthenia gravis: a case series in Germany. J Neurol. 2018;265(12):2773–82. 30225725 10.1007/s00415-018-9056-8

[71] Sacca F, Salort-Campana E, Jacob S, Cortes-Vicente E, Schneider-Gold C. Refocusing generalized myasthenia gravis: patient burden, disease profiles, and the role of evolving therapy. Eur J Neurol. 2024;31(6):e16180. 38117543 10.1111/ene.16180 PMC11236062

[72] Smulowitz PB, Zeller J, Sanchez LD, Edlow JA. Myasthenia gravis: lessons for the emergency physician. Eur J Emerg Med. 2005;12:324–26. 16276268 10.1097/00063110-200512000-00017

[73] Stetefeld H, Schroeter M. Sop myasthenic crisis. Neurol Res Pract. 2019;1:19. 33324885 10.1186/s42466-019-0023-3 PMC7650067

[74] Sivadasan A, Ma C-L, Cortel-LeBlanc A, Katzberg H. Peripheral nervous system and neuromuscular disorders in the emergency department: a review. Acad Emerg Med. 2024;31(4):386–97. 38419365 10.1111/acem.14861

[75] Wiendl H, Abicht A, Chan A, Della Marina A, Hagenacker T, Hekmat K, et al. Guideline for the management of myasthenic syndromes. Ther Adv Neurol Disord. 2023;16:17562864231213240. 38152089 10.1177/17562864231213240 PMC10752078

[76] Hillmen P, Lewis SM, Bessler M, Luzzatto L, Dacie JV. Natural history of paroxysmal nocturnal hemoglobinuria. N Engl J Med. 1995;333(19):1253–58. 7566002 10.1056/NEJM199511093331904

[77] Roth A, Maciejewski J, Nishimura JI, Jain D, Weitz JI. Screening and diagnostic clinical algorithm for paroxysmal nocturnal hemoglobinuria: expert consensus. Eur J Haematol. 2018;101(1):3–11. 29532535 10.1111/ejh.13059

[78] Schrezenmeier H, Muus P, Socie G, Szer J, Urbano-Ispizua A, Maciejewski JP, et al. Baseline characteristics and disease burden in patients in the international paroxysmal nocturnal hemoglobinuria registry. Haematologica. 2014;99(5):922–29. 24488565 10.3324/haematol.2013.093161 PMC4008114

[79] Richards SJ, Painter D, Dickinson AJ, Griffin M, Munir T, Arnold L, et al. The incidence and prevalence of patients with paroxysmal nocturnal haemoglobinuria and aplastic anaemia PNH syndrome: a retrospective analysis of the UK’s population-based haematological malignancy research network 2004-2018. Eur J Haematol. 2021;107(2):211–18. 34060690 10.1111/ejh.13640

[80] Takeda J, Miyata T, Kawagoe K, Iida Y, Endo Y, Fujita T, et al. Deficiency of the gpi anchor caused by a somatic mutation of the PIG-A gene in paroxysmal nocturnal hemoglobinuria. Cell. 1993;73(4):703–11. 8500164 10.1016/0092-8674(93)90250-t

[81] Socie G, Mary JY, de Gramont A, Rio B, Leporrier M, Rose C, et al. Paroxysmal nocturnal haemoglobinuria: long-term follow-up and prognostic factors. Fr Soc of Haematol. Lancet. 1996;348(9027):573–77. 10.1016/s0140-6736(95)12360-1 8774569

[82] Parker C, Omine M, Richards S, Nishimura J, Bessler M, Ware R, et al. Diagnosis and management of paroxysmal nocturnal hemoglobinuria. Blood. 2005;106(12):3699–709. 16051736 10.1182/blood-2005-04-1717 PMC1895106

[83] Luzzatto L, Gianfaldoni G, Notaro R. Management of paroxysmal nocturnal haemoglobinuria: a personal view. Br J Haematol. 2011;153(6):709–20. 21517820 10.1111/j.1365-2141.2011.08690.x

[84] Socié G, Bagou G. Orphanet emergency guidelines. Paroxysmal nocturnal haemoglobinuria. 2008. https://www.orpha.net/pdfs/data/patho/Pro/en/Emergency_ParoxysmalNocturnalHaemoglobinuria.pdf. Accessed 19 April 2025.

[85] Kremer Hovinga JA, Coppo P, Lammle B, Moake JL, Miyata T, Vanhoorelbeke K. Thrombotic thrombocytopenic purpura. Nat Rev Dis Primers. 2017;3:17020. 28382967 10.1038/nrdp.2017.20

[86] Mansouri Taleghani M, von Krogh as, Fujimura Y, George JN, Hrachovinova I, Knobl PN, et al. Hereditary thrombotic thrombocytopenic purpura and the hereditary TTP registry. Hamostaseologie. 2013;33(2):138–43. 23715103 10.5482/HAMO-13-04-0026

[87] Miesbach W, Menne J, Bommer M, Schonermarck U, Feldkamp T, Nitschke M, et al. Incidence of acquired thrombotic thrombocytopenic purpura in Germany: a hospital level study. Orphanet J Rare Dis. 2019;14(1):260. 31730475 10.1186/s13023-019-1240-0 PMC6858672

[88] Scully M, Cataland S, Coppo P, de la Rubia J, Friedman KD, Kremer Hovinga J, et al. Consensus on the standardization of terminology in thrombotic thrombocytopenic purpura and related thrombotic microangiopathies. J Thromb Haemost. 2017;15(2):312–22. 27868334 10.1111/jth.13571

[89] Long B, Bridwell RE, Manchanda S, Gottlieb M. Evaluation and management of thrombotic Thrombocytopenic Purpura in the emergency department. J Emerg Med. 2021;61(6):674–82. 34518045 10.1016/j.jemermed.2021.07.045

[90] Zheng XL, Vesely SK, Cataland SR, Coppo P, Geldziler B, Iorio A, et al. Good practice statements (GPS) for the clinical care of patients with thrombotic thrombocytopenic purpura. J Thromb Haemost. 2020;18(10):2503–12. 32914535 10.1111/jth.15009 PMC7880820

[91] Sadler JE. Pathophysiology of thrombotic thrombocytopenic purpura. Blood. 2017;130(10):1181–88. 28768626 10.1182/blood-2017-04-636431 PMC5606001

[92] Scully M, Antun A, Cataland SR, Coppo P, Dossier C, Biebuyck N, et al. Recombinant ADAMTS13 in congenital thrombotic Thrombocytopenic Purpura. N Engl J Med. 2024;390(17):1584–96. 38692292 10.1056/NEJMoa2314793

[93] Zheng XL, Vesely SK, Cataland SR, Coppo P, Geldziler B, Iorio A, et al. Isth guidelines for the diagnosis of thrombotic thrombocytopenic purpura. J Thromb Haemost. 2020;18(10):2486–95. 32914582 10.1111/jth.15006 PMC8146131

[94] Munoz NG, Ortega S, Solanich X, Cid J, Diaz M, Moreno AB, et al. Diagnosis and clinical management of thrombotic thrombocytopenic purpura (TTP): a consensus statement from the TTP Catalan group. Blood Transfus. 2024;22(2):176–84. 37677097 10.2450/BloodTransfus.522 PMC10920070

[95] Grant MJ, Booth A. A typology of reviews: an analysis of 14 review types and associated methodologies. Health Info Libr J. 2009;26(2):91–108. 19490148 10.1111/j.1471-1842.2009.00848.x

[96] Munn Z, Moola S, Lisy K, Riitano D, Tufanaru C. Methodological guidance for systematic reviews of observational epidemiological studies reporting prevalence and cumulative incidence data. Int J Evid Based Healthc. 2015;13(3):147–53. 26317388 10.1097/XEB.0000000000000054

[97] Ouzzani M, Hammady H, Fedorowicz Z, Elmagarmid A. Rayyan-a web and mobile app for systematic reviews. Syst Rev. 2016;5(1):210. 27919275 10.1186/s13643-016-0384-4 PMC5139140

[98] Fabiano N, Gupta A, Bhambra N, Luu B, Wong S, Maaz M, et al. How to optimize the systematic review process using ai tools. JCPP Adv. 2024;4(2):e12234. 38827982 10.1002/jcv2.12234 PMC11143948

[99] Haddaway NR, Page MJ, Pritchard CC, McGuinness LA. PRISMA2020: an R package and shiny app for producing prisma 2020-compliant flow diagrams, with interactivity for optimised digital transparency and open synthesis Campbell systematic reviews. 2022;18(e1230) 10.1002/cl2.1230 PMC8958186 36911350

[100] Kochar D, Pal M, Kochar S, Vyas A, Kochar A, Bindal D, et al. Acute intermittent porphyria presenting with neurological emergency: review of six cases. Neurol India. 2007;55(4):413–15. 18040123 10.4103/0028-3886.33303

[101] Bull TP, McCulloch R, Nicolson PLR, Doyle AJ, Shaw RJ, Langridge A, et al. Diagnostic uncertainty presented barriers to the timely management of acute thrombotic thrombocytopenic purpura in the United Kingdom between 2014 and 2019. J Thromb Haemostasis. 2022;20:1428–36. 35189012 10.1111/jth.15681 PMC9314944

[102] Korkmaz S, Keklik M, Sivgin S, Yildirim R, Tombak A, Kaya ME, et al. Therapeutic plasma exchange in patients with thrombotic thrombocytopenic purpura: a retrospective multicenter study. Tranfus And Apheresis Sci. 2013;48(3):353–58. 10.1016/j.transci.2013.04.016 23602056

[103] Stella CL, Dacus J, Guzman E, Dhillon P, Coppage K, How H, et al. The diagnostic dilemma of thrombotic thrombocytopenic purpura/hemolytic uremic syndrome in the obstetric triage and emergency department: lessons from 4 tertiary hospitals. Am J Obstet Gynecol. 2009;200:e1–381. 10.1016/j.ajog.2008.10.037 19110215

[104] Yang J, Chen Q, Yang H, Hua B, Zhu T, Zhao Y, et al. Clinical and laboratory features of acute porphyria: a study of 36 subjects in a Chinese tertiary referral center. Biomed Res Int. 2016;2016(no pagination) 10.1155/2016/3927635 PMC5153496 28025645

[105] Liu YP, Lien WC, Fang CC, Lai TI, Chen WJ, Wang HP. Ed presentation of acute porphyria. Am J Emerg Med. 2005;23(2):164–67. 15765337 10.1016/j.ajem.2004.03.013

[106] Kumar S, Sharma N, Modi M, Sharma A, Mahi S, Varma S. Spectrum of emergency department presentation in patients of acute intermittent porphyria: experience from a North Indian tertiary care center. Neurol India. 2010;58(1):95–98. 20228472 10.4103/0028-3886.60410

[107] Huseyin C, Aykac Cebicci M, Sahan M, Gurbuz S, Karaca B, Karakus A, et al. Triggers for attacks in familial mediterranean fever: are there any regional or ethnic differences? Acta Medica Mediterr. 2014;30:1349–53.

[108] Day C, Van der Walt J, Crombie K, Hendrikse C, Peter J. Acute angioedema in Cape Town emergency centres and a suggested algorithm to simplify and improve management. S Afr Med J. 2023;113(8):51–57. 37882115 10.7196/SAMJ.2023.v113i8.717

[109] Felder S, Curtis RM, Ball I, Borici-Mazi R. Prognostic factors in outcome of angioedema in the emergency department. Allergy Asthma Proc. 2014;35(5):362–70. 25295803 10.2500/aap.2014.35.3787

[110] Javaud N, Gompel A, Bouillet L, Boccon-Gibod I, Cantin D, Smaiti N, et al. Factors associated with hospital admission in hereditary angioedema attacks: a multicenter prospective study. Ann of Allergy, Asthma, Immunol. 2015;114(6):499–503. 25935434 10.1016/j.anai.2015.04.005

[111] Moldovan D, Bara N, Nadasan V, Gabos G, Mihaly E. Consequences of misdiagnosed and mismanaged hereditary angioedema laryngeal attacks: an overview of cases from the Romanian registry. Case Rep Emerg Med Print. 2018; 2018;6363787. 10.1155/2018/6363787 PMC6217881 30425862

[112] Pekdemir M, Ersel M, Aksay E, Yanturali S, Akturk A, Kiyan S. Effective treatment of hereditary angioedema with fresh frozen plasma in an emergency department. J Emerg Med. 2007;33(2):137–39. 17692764 10.1016/j.jemermed.2007.02.024

[113] Songur Kodik M, Inci O, Cetin ZD, Mete Gokmen EN, Karbek Akarca F. Evaluation of the retrospective lace index in predicting the risk of readmission in patients with hereditary angioedema in an emergency department. Emerg Med Int Print. 2023;2023:8847030. 10.1155/2023/8847030 PMC10611537 37900718

[114] Kuwayama K, Takase K, Kashihara M, Hondo H, Shigekiyo T, Takimoto O, et al. Central nervous system lesions associated with hereditary hemorrhagic telangiectasia–three case reports. Neurologia medico-chirurgica. 2003;43(9):447–51. 14560850 10.2176/nmc.43.447

[115] Boisriou I, Ellouze S, Kassasseya C, Feral-Pierssens AL, Gerlier C, Chauvin A, et al. Misdiagnosis of thrombotic microangiopathy in the emergency department: a multicenter retrospective study. Intern Emerg Med. 2023;1. 10.1007/s11739-023-03457-8 37914919

[116] Li XM, Mo XY, Huang GQ, Zhang FJ. Therapeutical plasma exchange for thrombotic thrombocytopenic purpura in the emergency department: a single center experience. Am J Emerg Med. 2021;46:556–59. 33214018 10.1016/j.ajem.2020.11.019

[117] Noel N, Maigne G, Tertian G, Anguel N, Monnet X, Michot JM, et al. Hemolysis and schistocytosis in the emergency department: consider pseudothrombotic microangiopathy related to vitamin B12 deficiency. Qjm. 2013;106:1017–22. 23842487 10.1093/qjmed/hct142

[118] Pieralli F, Mancini A, Camaiti A, Berni G, Nozzoli C. The ability of clinical and laboratory findings to predict in-hospital death in patients with thrombotic thrombocytopenic purpura in an internal and emergency medicine department. Italian J Med. 2011;5:269–73.

[119] Goyal V, Behari M, Singh S, Srivastava T. Myasthenic crisis: a retrospective study. Neurol India. 2004;52:453–56. 15626832

[120] Hsu CW, Chen NC, Huang WC, Lin HC, Tsai WC, Huang CC, et al. Hemogram parameters can predict in-hospital mortality of patients with myasthenic crisis. BMC Neurol. 2021;21. 10.1186/s12883-021-02412-4 PMC8493047 34615473

[121] Kim HJ, Choi JY, Yang HK, Hwang JM, Kim JS. Diplopia: characteristics and etiologic distribution in a referral-based university hospital. J Neurol. 2023;270:1067–75. 36355187 10.1007/s00415-022-11471-7

[122] Kumar N, Kaur S, Raj S, Lal V, Sukhija J. Causes and outcomes of patients presenting with Diplopia: a hospital-based study. Neuro-Ophthalmology. 2021;45:238–45. 34366511 10.1080/01658107.2020.1860091 PMC8312586

[123] Kumar S. Acute onset binocular diplopia: a retrospective observational study of 100 consecutive cases managed at a tertiary eye centre in Saudi Arabia. Eye (Basingstoke). 2020;34:1608–13. 10.1038/s41433-019-0705-7 PMC7608375 31801968

[124] El Zahran T, El Hadi D, Mostafa H, Mansour H, Hashim I, Tahhan S, et al. The yield of neuroimaging in patients presenting to the emergency department with isolated neuro-ophthalmological complaints: a retrospective chart review. Med (U States). 2023;102:E32740. 10.1097/MD.0000000000032740 PMC9875971 36705369

[125] Al-Attar M, Butterworth S, McKay L. A quantitative and qualitative analysis of patient group narratives suggests common biopsychosocial red flags of undiagnosed rare disease. Orphanet J Rare Dis. 2024;19(1):172. 38641814 10.1186/s13023-024-03143-8 PMC11031885

[126] Grigull L. Die richtige Recherche bis zur Diagnose [The right research until diagnosis]. In: Mücke M, Conrad R, editors. Elsevier Essentials seltene Erkrankungen: das Wichtigste für Ärztinnen und Ärzte aller Fachrichtungen [Elsevier Essentials rare diseases: the most important information for doctors of all specialities]. Munich, Germany: Elsevier: Urban-&-Fischer-Verlag; 2021. p. 31–34.

