# Supplementary material for: Common features of rare disease patients in the emergency department: a systematised literature review
Source: Orphanet J Rare Dis. 2025 Nov 13;20:582. doi: 10.1186/s13023-025-04111-6 (PMC12616910; doi:10.1186/s13023-025-04111-6)
Supplement: Supplementary file 1 — Supplementary Material 1 [file 13023_2025_4111_MOESM1_ESM.docx]

SUPPLEMENTARY Material 1

|  | Embase | MEDLINE | Web of  Science | Google Scholar | Cochrane  Library |
| --- | --- | --- | --- | --- | --- |
| AHP | 1 “porphyria*”.ab,ti.  2 “acute porphyria*”.ab,ti.  3 “porphyrin disorder*”.ab,ti.  4 porphyria/ or porphyria variegata/ or acute intermittent porphyria/ or hepatic porphyria/  5 “ED presentation*”.ab,ti.  6 “emergency department presentation*”.ab,ti.  7 “hospital emergency service*”.ab,ti.  8 “emergency care”.ab,ti.  9 “emergency health service*”.ab,ti.  10 “emergency treatment”.ab,ti.  11 “emergency ward”.ab,ti.  12 “emergency medicine”.ab,ti.  13 “emergency care”.ab,ti.  14 “emergency unit*”.ab,ti.  15 ”emergency room”.ab,ti.  16 “emergency”.ab,ti,in.  17 “emergency patient*”.ab,ti.  18 ”emergency department*”.ab,ti,in.  19 emergency ward/  20 emergency medicine/  21 emergency physician/  22 “emergency physician*”.ab,ti.  23 1 or 2 or 3 or 4  24 5 or 6 or 7 or 8 or 9 or 10 or 11 or 12 or 13 or 14 or 15 or 16 or 17 or 18 or 19 or 20 or 21 or 22  25 23 and 24 | 1 Emergency Service, Hospital/  2 “emergency treatment*”.ab,ti.  3 “emergency health service*”.ab,ti.  4 “emergency patient*”.ab,ti.  5 "hospital emergency service*".ab,ti.  6 “emergency care”.ab,ti.  7 “emergency ward”.ab,ti.  8 “emergency medicine”.ab,ti.  9 Emergency Medicine/  10 "ED presentation*".ab,ti.  11 "emergency department presentation*".ab,ti.  12 "emergency unit*".ab,ti.  13 "emergency room".ab,ti.  14 “emergency”.ab,ti,in.  15 "emergency department".ab,ti,in.  16 “emergency physician*”.ab,ti.  17 1 or 2 or 3 or 4 or 5 or 6 or 7 or 8 or 9 or 10 or 11 or 12 or 13 or 14 or 15 or 16  18 "porphyria*".ab,ti.  19 "acute porphyria*".ab,ti.  20 "porphyrin disorder*".ab,ti.  21 Porphyrias/  22 18 or 19 or 20 or 21  23 17 and 22 | (TS=(ED presentation*) OR TS=(emergency department presentation*) OR TS=(hospital emergency service*) OR TS=(emergency care) OR TS=(emergency health service*) OR TS=(emergency treatment) OR TS=(emergency ward) OR TS=(emergency medicine) OR TS=(emergency unit*) OR TS=(emergency room) OR TS=(emergency) OR TS=(emergency patient*) OR TS=(emergency department*) OR TS=(emergency physician*)) AND (TS=(porphyria*) OR TS=(acute porphyria*) OR TS=(porphyrin disorder*)) | 1.) allintitle: "porphyria*" "ED presentation*" OR "emergency department presentation*" OR "emergency health service*" OR "hospital emergency service*" OR "emergency care" OR "emergency treatment" OR "emergency ward" OR "emergency medicine" OR "emergency care" OR "emergency unit*" OR "emergency room" OR "emergency" OR "emergency patient*" OR "emergency department*" OR "emergency physician*"  2.) allintitle: "acute porphyria*" "ED presentation*" OR "emergency department presentation*" OR "emergency health service*" OR "hospital emergency service*" OR "emergency care" OR "emergency treatment" OR "emergency ward" OR "emergency medicine" OR "emergency care" OR "emergency unit*" OR "emergency room" OR "emergency" OR "emergency patient*" OR "emergency department*" OR "emergency physician*" | 1 ED presentation*:ti,ab,kw  2 emergency department presentation*:ti,ab,kw  3 emergency health service:ti,ab,kw  4 hospital emergency sevice:ti,ab,kw  5 emergency care:ti,ab,kw  6 emergency treatment:ti,ab,kw  7 emergency ward:ti,ab,kw  8 emergency medicine:ti,ab,kw  9 emergency care:ti,ab,kw  10 emergency unit*:ti,ab,kw  11 emergency room:ti,ab,kw  12 emergency:ti,ab,kw  13 emergency patient*:ti,ab,kw  14 emergency department*:ti,ab,kw  15 ("emergency physician"):ti,ab,kw  16 MeSH descriptor: [Emergency Service, Hospital] this term only  17 MeSH descriptor: [Emergency Medicine] this term only  18 1 or 2 or 3 or 4 or 5 or 6 or 7 or 8 or 9 or 10 or 11 or 12 or 13 or 14 or 15 or 16 or 17  19 (porphyria*):ti,ab,kw  20 (acute porphyria*):ti,ab,kw  21 (porphyrin disorder*):ti,ab,kw  22 MeSH descriptor: [Porphyrias] this term only  23 19 or 20 or 21 or 22  24 18 and 23 |

|  | Embase | MEDLINE |
| --- | --- | --- |
| Emergency Department | 1 "ED presentation*".ab,ti.  2 "emergency department presentation*".ab,ti.  3 "hospital emergency service*".ab,ti.  4 “emergency care”.ab,ti.  5 "emergency health service*".ab,ti.  6 “emergency treatment”.ab,ti.  7 “emergency ward”.ab,ti.  8 “emergency medicine”.ab,ti.  9 "emergency unit*".ab,ti.  10 “emergency room”.ab,ti.  11 “emergency”.ab,ti,in.  12 "emergency patient*".ab,ti.  13 "emergency department*".ab,ti,in.  14 emergency ward/  15 emergency medicine/  16 emergency physician/  17 emergency physician*.ab,ti.  18 1 or 2 or 3 or 4 or 5 or 6 or 7 or 8 or 9 or 10 or 11 or  12 or 13 or 14 or 15 or 16 or 17 | 1 Emergency Service, Hospital/  2 "emergency treatment*".ab,ti.  3 "emergency health service*".ab,ti.  4 "emergency patient*".ab,ti.  5 "hospital emergency service*".ab,ti.  6 “emergency care”.ab,ti.  7 “emergency ward”.ab,ti.  8 “emergency medicine”.ab,ti.  9 Emergency Medicine/  10 "ED presentation*".ab,ti.  11 "emergency department presentation*".ab,ti.  12 "emergency unit*".ab,ti.  13 "emergency room".ab,ti.  14 “emergency”.ab,ti,in.  15 "emergency department".ab,ti,in.  16 “emergency physician*”.ab,ti.  17 1 or 2 or 3 or 4 or 5 or 6 or 7 or 8 or 9 or 10 or 11 or 12 or 13 or  14 or 15 or 16 |
| HAE | 1-18 = search strategy for ED  19 "C1-INH deficiency".ab,ti.  20 "C1 Inhibitor Deficiency".ab,ti.  21 "hereditary angioneurotic edema*".ab,ti.  22 "hereditary angioneurotic oedema*".ti,ab.  23 "C1 Esterase Inhibitor Deficiency".ab,ti.  24 "hereditary angiooedema*".ti,ab.  25 "hereditary angioedema*".ti,ab.  26 19 or 20 or 21 or 22 or 23 or 24 or 25  27 18 and 26 | 1-17 = search strategy for ED  18 "C1-INH deficiency".ab,ti.  19 "C1 Inhibitor Deficiency".ab,ti.  20 "hereditary angioneurotic edema*".ab,ti.  21 "hereditary angioneurotic oedema*".ab,ti.  22 "C1 Esterase Inhibitor Deficiency".ab,ti.  23 "hereditary angiooedema*".ab,ti.  24 "hereditary angioedema*".ab,ti.  25 Angioedemas, Hereditary/  26 18 or 19 or 20 or 21 or 22 or 23 or 24 or 25  27 17 and 26 |
| MG | 1-18 = search strategy for ED  19 myasthenia gravis.ab,ti.  20 myasthen*.ab,ti.  21 myasthen* crisis.ab,ti.  22 myasthenia gravis/  23 19 or 20 or 21 or 22  24 18 and 23 | 1-17 = search strategy for ED  18 "myasthenia gravis".ab,ti.  19 myasthen*.ab,ti.  20 "myasthen* crisis".ab,ti.  21 Myasthenia Gravis/  22 18 or 19 or 20 or 21  23 17 and 22 |
| FMF | 1-18 = search strategy for ED  19 "familial mediterranean fever".ab,ti.  20 FMF.ab,ti.  21 "Mediterranean familial fever".ab,ti.  22 "periodic disease".ab,ti.  23 "periodic fever".ab,ti.  24 "Recurrent Polyserositis".ab,ti.  25 "Familial Paroxysmal Polyserositis".ab,ti.  26 "periodic peritonitis".ab,ti.  27 familial Mediterranean fever/  28 19 or 20 or 21 or 22 or 23 or 24 or 25 or 26 or 27  29 18 and 28 | 1-17 = search strategy for ED  18 "familial mediterranean fever".ab,ti.  19 FMF.ab,ti.  20 "Mediterranean familial fever".ab,ti.  21 "periodic disease".ab,ti.  22 "periodic fever".ab,ti.  23 "Recurrent Polyserositis".ab,ti.  24 "Familial Paroxysmal Polyserositis".ab,ti.  25 "periodic peritonitis".ab,ti.  26 Familial Mediterranean Fever/  27 18 or 19 or 20 or 21 or 22 or 23 or 24 or 25 or 26  28 17 and 27 |
| TTP | 1-18 = search strategy for ED  19 "Thrombotic Thrombocytopenic Purpura".ab,ti.  20 TTP.ab,ti.  21 "Moschkowitz Disease".ab,ti.  22 "Moschcowitz Disease".ab,ti.  23 "Upshaw Schulman Syndrome".ab,ti.  24 thrombotic thrombocytopenic purpura/  25 19 or 20 or 21 or 22 or 23 or 24  26 18 and 25 | 1-17 = search strategy for ED  18 "Thrombotic Thrombocytopenic Purpura".ab,ti.  19 TTP.ab,ti.  20 "Moschkowitz Disease".ab,ti.  21 "Moschcowitz Disease".ab,ti.  22 "Upshaw Schulman Syndrome".ab,ti.  23 Purpura, Thrombotic Thrombocytopenic/  24 18 or 19 or 20 or 21 or 22 or 23  25 17 and 24 |
| HHT | 1-18 = search strategy for ED  19 "Osler Weber Rendu Syndrome".ab,ti.  20 "HHT".ab,ti.  21 "Weber Osler Disease".ab,ti.  22 "Osler Rendu Disease".ab,ti.  23 "Osler's Disease".ab,ti.  24 "Hereditary Hemorrhagic Telangiectasia".ab,ti.  25 "Osler Disease".ab,ti.  26 "Osler Rendu Weber Disease".ab,ti.  27 "Weber Osler Syndrome".ab,ti.  28 "Morbus Osler".ab,ti.  29 19 or 20 or 21 or 22 or 23 or 24 or 25 or 26 or 27 or 28  30 18 and 29 | 1-17 = search strategy for ED  18 "Osler Weber Rendu Syndrome".ab,ti.  19 HHT.ab,ti.  20 "Weber Osler Disease".ab,ti.  21 "Osler Rendu Disease".ab,ti.  22 "Osler's Disease".ab,ti.  23 "Hereditary Hemorrhagic Telangiectasia".ab,ti.  24 "Osler Disease".ab,ti.  25 "Osler Rendu Weber Disease".ab,ti.  26 "Weber Osler Syndrome".ab,ti.  27 "Morbus Osler".ab,ti.  28 Telangiectasia, Hereditary Hemorrhagic/  29 18 or 19 or 20 or 21 or 22 or 23 or 24 or 25 or 26 or 27 or 28  30 17 and 29 |
| PNH | 1-18 = search strategy for ED  19 PNH.ab,ti.  20 "Paroxysmal Nocturnal Hemoglobinuria".ab,ti.  21 "Paroxysmal Hemoglobinuria".ab,ti.  22 "Marchiafava-Micheli Syndrome".ab,ti.  23 paroxysmal nocturnal hemoglobinuria/  24 19 or 20 or 21 or 22 or 23  25 18 and 24 | 1-17 = search strategy for ED  18 PNH.ab,ti.  19 "Paroxysmal Nocturnal Hemoglobinuria".ab,ti.  20 "Paroxysmal Hemoglobinuria".ab,ti.  21 "Marchiafava-Micheli Syndrome".ab,ti.  22 Hemoglobinuria, Paroxysmal/  23 18 or 19 or 20 or 21 or 22  24 17 and 23 |
| FD | 1-18 = search strategy for ED  19 Fabry.ab,ti.  20 "Morbus Fabry".ab,ti.  21 "fabry disease*".ab,ti.  22 "alpha galactosidase a deficiency".ab,ti.  23 "Anderson Fabry disease".ab,ti  24 FD.ab,ti.  25 Fabry disease/  26 alpha galactosidase/  27 19 or 20 or 21 or 22 or 23 or 24 or 25 or 26  28 18 and 27 | 1-17 = search strategy for ED  18 Fabry.ab,ti.  19 "Morbus Fabry".ab,ti.  20 "fabry disease*".ab,ti.  21 "alpha galactosidase a deficiency".ab,ti.  22 "Anderson Fabry disease".ab,ti.  23 FD.ab,ti.  24 Fabry Disease/  25 alpha-Galactosidase/  26 18 or 19 or 20 or 21 or 22 or 23 or 24 or 25  27 17 and 26 |
