# Supplementary material for: Common features of rare disease patients in the emergency department: a systematised literature review
Source: Orphanet J Rare Dis. 2025 Nov 13;20:582. doi: 10.1186/s13023-025-04111-6 (PMC12616910; doi:10.1186/s13023-025-04111-6)
Supplement: Supplementary file 2 — Supplementary Material 2 [file 13023_2025_4111_MOESM2_ESM.docx]

SUPPLEMENTARY Material 2

**AHP**

**Fulltext-Screening (n=12):**

| **Publication** | **Inclusion/**  **Exclusion** | **Reason for Exclusion** |
| --- | --- | --- |
| Bizovi and Emerson 2000 | E | Wrong publication type: Case report |
| Dombeck and Satonik 2005 | E | Wrong publication type: Review |
| Edel et al. 2023 | E | Wrong outcome/setting: Topic not symptoms and characteristics of patients with acute porphyria in ED |
| Gasperini et al. 2016 | E | Wrong publication type: Case report |
| Kochar et al. 2007 | E | Wrong outcome/setting: Setting not defined → not ED |
| Kostrzewska et al. 1974 | E | Wrong outcome/setting: Setting not ED |
| Kumar et al. 2010 | I |  |
| Liu et al. 2005 | I |  |
| Murali and El Hayek 2021 | E | Wrong publication type: Review |
| Yang et al. 2016 | I |  |
| Yang et al. 2018 | E | Wrong outcome/setting: Outcome measures not clinical description of patients with AHP in ED |
| Young et al. 2020 | E | Wrong publication type: Case report |

🡺 Included = 3

**HAE**

**Fulltext-Screening (n=60):**

| **Publication** | **Inclusion/**  **Exclusion** | **Reason for Exclusion** |
| --- | --- | --- |
| Agostoni & Cicardi 2001 | E | Review |
| Aytekin et al. 2021 | E | Topic: Description of Symptom/Patient characteristics but - Setting not Emergency Department |
| Bafunno et al. 2014 | E | Outcome no description of HAE in ED |
| Betschel et al. 2020 | E | Review |
| Bork et al. 2003 | E | Topic: Description of Symptom/Patient characteristics but - Setting not Emergency Department |
| Bork et al. 2000 | E | Topic: no description of HAE-presentation in ED |
| Buhler et al. 2023 | E | Review |
| Burton et al. 2023 | E | Topic: Description of Symptom/Patient characteristics but - Setting not Emergency Department |
| Cao et al. 2021 | E | Topic: Description of Symptom/Patient characteristics but - Setting not Emergency Department |
| Castellano et al. 2018 | E | Outcome no description of HAE in ED |
| Cheng et al. 2007 | E | Case report |
| Cheng et al. 2020 | E | Topic: Setting ED – but no description of HAE related symptoms |
| Christiansen et al. 2023 | E | Topic: Description of Symptom/Patient characteristics but - Setting not Emergency Department |
| Coulier et al. 2004 | E | Outcome no description of HAE in ED |
| Craig et al. 2010 | E | Topic: no description of HAE-presentation in ED |
| Day et al. 2023 | I |  |
| de Wazieres et al. 1995 | E | Language not English or German |
| Felder et al. 2014 | I |  |
| Forjaz et al. 2020 | E | Topic: Description of Symptom/Patient characteristics but - Setting not Emergency Department |
| Fukunaga et al. 2022 | E | Topic: Survey of physicians on HAE |
| Gakhal & Marcotte 2015 | E | Review |
| Grumach et al. 2017 | E | Review |
| Gurmen et al. 2017 | E | Case report |
| Hermans et al. 2022 | E | Language not English or German |
| Hirose et al. 2017 | I |  |
| Honda et al. 2021 | E | Topic: no description of HAE-presentation in ED |
| Honda et al. 2023 | E | Wrong outcome measures - no description of HAE patient characteristics in ED |
| Iwamoto et al. 2011 | E | Language not English or German |
| Jaiganesh et al. 2012 | E | Topic: no description of HAE-presentation in ED |
| Javaud et al. 2013 | E | Topic: no description of HAE-presentation in ED |
| Javaud et al. 2015 | I |  |
| Javaud et al. 2015b | E | Outcome no description of HAE in ED |
| Javaud et al. 2016 | E | Wrong Outcome measure |
| Javaud et al. 2019 | E | Topic: Description of Symptom/Patient characteristics but - Setting not Emergency Department |
| Karadza-Lapic et al. 2018 | E | Topic: no description of HAE-presentation in ED |
| Kedarisetty et al. 2021 | E | Topic: no description of HAE-presentation in ED |
| Longhurst & Bygum 2016 | E | Review |
| Moldovan et al. 2018 | I |  |
| Nunes et al. 2021 | E | Topic: no description of HAE-presentation in ED |
| Ohsawa et al. 2013 | E | Topic: no description of symptoms and clinical characteristics of HAE-presentation in ED |
| Otani et al. 2017 | E | Topic: no description of symptoms and clinical characteristics of HAE-presentation in ED |
| Pekdemir et al. 2007 | I |  |
| Pines et al. 2021 | E | Review |
| Riedl et al. 2018 | E | Topic: no description of symptoms and clinical characteristics of HAE-presentation in ED |
| Riguzzi et al. 2014 | E | Wrong study population (age < 16y) |
| Rosado-Quinones & Zaragoza-Urdaz 2019 | E | Setting not ED |
| Sastre Castillo et al. 1979 | E | Setting not ED |
| Serpa et al. 2021 | E | Review |
| Songur Kodik et al. 2023 | I |  |
| Squeglia et al. 2016 | E | Topic: no description of symptoms and clinical characteristics of HAE-presentation in ED |
| Tachdjian et al. 2020 | E | Setting not ED |
| Ucar et al. 2016 | E | Outcome: no sufficient description of symptoms and clinical characteristics of HAE-presentation in ED |
| Veronez et al. 2019 | E | Topic: no description of symptoms and clinical characteristics of HAE-presentation in ED |
| Wentzel et al. 2019 | E | Topic: no description of symptoms and clinical characteristics of HAE-presentation in ED |
| Wilkerson 2023 | E | Wrong outcome measures - no description of HAE patient characteristics in ED |
| Wilkerson & Moellman 2022 | E | Review |
| Wilkerson & Moellman 2023 | E | Review |
| Zhou et al. 2023 | E | Language not English or German |
| Zilberberg et al. 2010 | E | Topic: no description of symptoms and clinical characteristics of HAE-presentation in ED |
| Zilberberg et al. 2011 | E | Outcome: no sufficient description of symptoms and clinical characteristics of HAE-presentation in ED |

🡺 Included = 7

**MG**

**Fulltext-Screening (n= 33):**

| **Publication** | **Inclusion/**  **Exclusion** | **Reason for Exclusion** |
| --- | --- | --- |
| Bizovi and Emerson 2000 | E | Case report |
| Goyal et al. 2004 | E | Setting not ED (Description of 11 patients with myasthenic crisis) |
| Cheng et al. 2009 | E | Setting not ED + wrong outcome measures |
| Chung et al. 2021 | E | No exploration of symptoms and patient characteristics of MG-Patients in ED |
| Cutts and Morris 2001 | E | Case report → Wrong Report Type |
| De Meel et al. 2015 | E | No exploration of symptoms and patient characteristics of MG-Patients in ED |
| El Zahran et al. 2023 | E | No exploration of symptoms and patient characteristics of MG-Patients in ED |
| Farah et al. 2005 | E | Case report |
| Gummi et al. 2019 | E | No exploration of symptoms and patient characteristics of MG-Patients in ED |
| Gupta et al. 2009 | E | Case Report → Wrong Report Type |
| Hsu et al. 2021 | E | No exploration of symptoms and patient characteristics of MG-Patients in ED |
| Jacoby and Weathers 2022 | E | Wrong Report Type → CME Article |
| Jeong et al. 2021 | E | No exploration of symptoms and patient characteristics of MG-Patients in ED |
| Kennedy 1968 | E | Wrong Report Type → Review |
| Kim et al. 2023 | E | No exploration of symptoms and patient characteristics of MG-Patients in ED |
| Klein et al. 2020 | E | Wrong Report Type → Case report |
| Kumar et al. 2021 | E | No exploration of symptoms and patient characteristics of MG-Patients in ED |
| Kumar 2020 | E | No exploration of symptoms and patient characteristics of MG-Patients in ED |
| Kunze 2004 | E | Wrong Report Type → Review |
| Kutzin 2012 | E | Wrong Report Type → Case report |
| Layat et al. 2017 | E | No exploration of symptoms and patient characteristics of MG-Patients in ED |
| Matsuura and Sogabe 2019 | E | Wrong Report Type → Case report |
| Minagar et al. 2012 | E | Wrong Report Type → Case report |
| Moral et al. 2010 | E | No exploration of symptoms and patient characteristics of MG-Patients in ED |
| Noto and Marcolini 2014 | E | Wrong Report Type → Review |
| Nowakowski and Yee 1982 | E | Case report |
| Nwani et al. 2021 | E | No exploration of symptoms and patient characteristics of MG-Patients in ED |
| Samtleben et al. 1982 | E | No exploration of symptoms and patient characteristics of MG-Patients in ED |
| Santy et al. 2022 | E | Wrong Report Type → Case report |
| Sasaki et al. 2014 | E | Wrong Report Type → Case report |
| Smulowitz et al. 2005 | I |  |
| Trueger 2022 | E | No exploration of symptoms and patient characteristics of MG-Patients in ED |
| Viets 1946 | E | No exploration of symptoms and patient characteristics of MG-Patients in ED |

🡺 Included = 1

**FMF**

**Fulltext-Screening (n=18):**

| **Publication** | **Inclusion/**  **Exclusion** | **Reason for Exclusion** |
| --- | --- | --- |
| Becel et al. 2016 | E | Topic: no clinical characterization of FMF-patients in ED |
| Bentancur et al. 2005 | E | Topic: no clinical characterization of FMF-patients in ED |
| Breuer and Taurog 2014 | E | Wrong report type: Case report |
| Cebeci et al. 2023 | E | Outcome measures not description of FMF patients in ED |
| Celik et al. 2015 | E | Setting not ED/Outcome measures not description of FMF patients in ED |
| Ciftci et al. 1995 | E | Children |
| Erdem and Sultanoglu 2021 | E | Topic: no clinical characterization of FMF-patients in ED |
| Holl and Holl 2019 | E | Case report |
| Huseyin et al. 2014 | I |  |
| Jafari et al. 2018 | E | Topic: no clinical characterization of FMF-patients in ED |
| Kishida et al. 2018 | E | Setting not ED/Outcome measures not description of FMF patients in ED |
| Livneh et al. 1997 | E | Topic: no clinical characterization of FMF-patients in ED |
| Maconi et al. 2018 | E | Review |
| Masatlioglu et al. 2011 | E | Topic: no clinical characterization of FMF-patients in ED |
| Ocak et al. 2013 | E | Topic: no clinical characterization of FMF-patients in ED |
| San et al. 2020 | E | Topic: no clinical characterization of FMF-patients in ED |
| Sever et al. 2012 | E | Wrong language: turkish |
| Tanriverdi et al. 2022 | E | Topic: no clinical characterization of FMF-patients in ED |

🡺 Included = 1

**TTP**

**Fulltext-Screening (n=52 *+1 no abstract/full text available*):**

| **Publication** | **Inclusion/**  **Exclusion** | **Reason for Exclusion** |
| --- | --- | --- |
| Abdel Karim et al. 2013 | E | Wrong Outcome measures: no sufficient description of (initial) ED  presentation of patients with TTP |
| Berti de Marinis et al. 2016 | E | Wrong outcome - no description of TTP patients in ED |
| Bonoga et al. 2022 | E | Case report |
| Bull et al. 2022 | E | Setting not known → not ED |
| Caronna et al. 2005 | E | Wrong Outcome measures: no sufficient description of (initial) ED  presentation of patients with TTP |
| Coppo et al. 2016 | E | Wrong language |
| De Boisriou et al. 2023 | I |  |
| Dong et al. 2023 | E | Wrong Outcome measures: no sufficient description of (initial) ED  presentation of patients with TTP |
| Ellis et al. 2015 | E | Case Report |
| Farhat et al. 2011 | E | Wrong Outcome measures: no sufficient description of (initial) ED  presentation of patients with TTP (description of three cases with disseminated malignancy presenting as TMA) |
| Ganesh and Varma 2020 | E | Wrong study type: Letter |
| Gasparovic et al. 2000 | E | Wrong outcome - no description of TTP patients in ED |
| Habe et al. 2012 | E | Wrong outcome - no description of TTP patients in ED |
| Karoui et al. 2019 | E | Wrong Outcome measures: no sufficient description of (initial) ED  presentation of patients with TTP |
| Kemkes-Matthes et al. 1999 | E | Wrong study type: Review |
| Kessler et al. 2012 | E | Wrong study type: Review |
| Khatun and Morshed 2015 | E | Study design: setting not defined as ED |
| Kinnaman et al. 2015 | E | Wrong Study type: Case report |
| Korkmaz et al. 2013 | E | Setting not specified → Setting not ED |
| Lavender and Collin 2021 | E | Wrong Study type: Case report |
| Li et al. 2021 | I |  |
| Liferidge and  Dark 2013 | E | Wrong study type: Review |
| Liu et al. 2023 | E | Wrong outcome - no description of TTP patients in ED |
| Lim and Park 2016 | E | Wrong study type: case report |
| Mancio et al. 2015 | E | Setting not ED sondern Hämatologische Klinik |
| McCormick et al. 2007 | E | Wrong study type: case report |
| McSparron et al. 2017 | E | Wrong topic - no description of TTP patients in ED |
| Milon et al. 2022 | E | Wrong study type: case report |
| Nagajothi and Braverman 2007 | E | Wrong Outcome measures: no sufficient description of (initial) ED  presentation of patients with TTP |
| Nakamura et al. 2016 | E | Wrong Outcome measures: no sufficient description of (initial) ED  presentation of patients with TTP |
| Nishimura et al. 2023 | E | Setting not ED |
| Noel et al. 2013 | I |  |
| Page et al. 1991 | E | Wrong outcome - no description of TTP patients in ED |
| Peters et al. 2015 | E | Wrong study type: case report |
| Peterson et al. 1979 | E | Setting not Emergency Department |
| Pieralli et al. 2011 | I |  |
| Pourrat et al. 1994 | E | Wrong Outcome measures: no sufficient description of (initial) ED  presentation of patients with TTP |
| Rane et al. 2014 | E | Wrong study type: case report |
| Ryan et al. 1979 | E | Wrong Outcome measures: no sufficient description of (initial) ED  presentation of patients with TTP |
| Samaras et al. 2008 | E | Wrong Outcome measures: no sufficient description of (initial) ED  presentation of patients with TTP |
| Sawyer 2010 | E | Wrong study type: review |
| Scharrer 2005 | E | Wrong study type: review |
| Shiber and Fines 2011 | E | Wrong study type: case report |
| Shih et al. 2011 | E | Wrong study type: case report |
| Smith et al. 2019 | E | Wrong study type: case report |
| Soares et al. 2023 | E | Wrong Outcome measures: no sufficient description of (initial) ED  presentation of patients with TTP |
| Stella et al. 2009 | I |  |
| Sun et al. 2021 | E | Setting not clearly ED, not stated if patients had reported symptoms in ED or during hospital stay |
| Tardy et al. 1989 | E | Wrong study type: case report |
| Thongprayoon et al. 2015 | E | Wrong study type: case report |
| Wan et al. 2013 | E | Setting not ED |
| Wang et al. 2020 | E | Wrong outcome - no description of TTP patients in ED |
| Yue et al. 2020 | E | Wrong outcome - no description of TTP patients in ED |

🡺 Included = 5

**HHT**

**Fulltext-Screening (n=18):**

| **Publication** | **Inclusion/**  **Exclusion** | **Reason for Exclusion** |
| --- | --- | --- |
| Abangah and Rashidbeygi 2013 | E | Wrong study type: Case report |
| Alcala-Villalon et al. 2012 | E | Wrong language |
| Butt et al. 2016 | E | Wrong study type: Case report |
| Canders and Silman 2014 | E | Wrong study type: Case report |
| Felix et al. 2008 | E | Wrong study type: Case report |
| Gallitelli et al. 2006 | E | Outcome: no sufficient description of HHT-patient presentation in ED |
| Hauswald et al. 1986 | E | Outcome: no sufficient description of HHT-patient presentation in ED |
| Hu et al. 2009 | E | Wrong study type: Case report |
| Irani and Kasmani 2009 | E | Wrong study type: Case report |
| Klepchick and McLean 2006 | E | Wrong study type: Case report |
| Kuhajda et al. 2015 | E | Outcome: no sufficient description of HHT-patient presentation in ED (description of 4 patients with pulmonary pAVMs) |
| Kuwayama et al. 2003 | I |  |
| Pavel et al. 2022 | E | Setting not ED |
| Popovic et al. 2019 | E | Setting not ED |
| Purkey et al. 2014 | E | Outcome: no sufficient description of HHT-patient presentation in ED |
| Stoddard et al. 2014 | E | Outcome: no sufficient description of HHT-patient presentation in ED |
| Sueda et al. 2020 | E | Case report |
| Vaiman et al. 2004 | E | Outcome: no sufficient description of HHT-patient presentation in ED |

🡺 Included = 1

**PNH**

**Fulltext-Screening (n=5):**

| **Publication** | **Inclusion/**  **Exclusion** | **Reason for Exclusion** |
| --- | --- | --- |
| Berlin and Queen 2002 | E | Wrong study type: Case report |
| Cavallaro et al. 2022 | E | Wrong study type: Case report |
| Lee 1973 | E | Wrong study type: Case report |
| Mathew et al. 2022 | E | Outcome: no sufficient description of PNH-patient presentation in ED |
| Ninan et al. 2023 | E | Wrong study type: Case report |

🡺 Included = 0

**FD**

**Fulltext-Screening (n=16):**

| **Publication** | **Inclusion/**  **Exclusion** | **Reason for Exclusion** |
| --- | --- | --- |
| Benz et al. 2019 | E | Outcome: no sufficient description of Fabry-patient presentation/characteristics in ED |
| Bersano et al. 2012 | E | Review |
| Celic et al. 2022 | E | Outcome: no sufficient description of Fabry-patient presentation/characteristics in ED |
| Fox 2012 | E | Outcome: no sufficient description of Fabry-patient presentation/characteristics in ED |
| Khan and Rasool 2021 | E | Outcome: no sufficient description of Fabry-patient presentation/characteristics in ED |
| Lillo et al. 2022 | E | Outcome: no sufficient description of Fabry-patient presentation/characteristics in ED |
| Militaru et al. 2018 | E | Review |
| Meucci et al. 2023 | E | Outcome: no sufficient description of Fabry-patient presentation/characteristics in ED |
| Militaru et al. 2019a | E | Case Report |
| Militaru et al. 2019b | E | Outcome: no sufficient description of Fabry-patient presentation/characteristics in ED |
| Militaru et al. 2019c | E | Outcome: no sufficient description of Fabry-patient presentation/characteristics in ED |
| Nouh et al. 2014 | E | Outcome: no sufficient description of Fabry-patient presentation/characteristics in ED |
| Oli et al. 2023 | E | Outcome: no sufficient description of Fabry-patient presentation/characteristics in ED |
| Sene et al. 2016 | E | Outcome: no sufficient description of Fabry-patient presentation/characteristics in ED |
| Sestito et al. 2020 | E | Review |
| Visoiu et al. 2019 | E | Case report |

🡺 Included = 0
